# Use of Wild Edible Plants: Can They Meet the Dietary and Nutritional Needs of Indigenous Communities in Central India

**DOI:** 10.3390/foods10071453

**Published:** 2021-06-23

**Authors:** Alka Mishra, Singam Laxmana Swamy, Tarun Kumar Thakur, Rajeev Bhat, Arvind Bijalwan, Amit Kumar

**Affiliations:** 1 Department of Rural Technology, Guru Ghasidas Central University, Bilaspur 495 001, India; alkamishra142@gmail.com; 2 College of Agriculture, Indira Gandhi Agricultural University, Katghora-Korba 495 445, India; singamswamy66@gmail.com; 3 Department of Environmental Science, Indira Gandhi National Tribal University, Amarkantak 484 887, India; 4 Estonian University of Life Sciences, Fr. R. Kreutzwaldi 1a, 51006 Tartu, Estonia; rajeev.bhat@emu.ee; 5 College of Forestry, VCSG Uttarakhand University of Horticulture & Forestry, Ranichauri 249 199, India; arvindbijalwan276@gmail.com; 6 School of Hydrology and Water Resources, Nanjing University of Information Science and Technology, Nanjing 210044, China

**Keywords:** biodiversity, food security, ecosystem services, Sustainable Development Goals (SDG), wild edible plants

## Abstract

Despite significant evidence base on quantifying ecosystem services, the role of biodiversity in supporting such services in diversified landscapes, and how indigenous communities exploit, utilize and manage plant resources in a biocultural regime, remains understudied. This study examines the role of wild edible plants (WEPs) in meeting the food, nutrition and household income of indigenous communities under the biodiversity rich landscape of the Achanakmaar-Amarkantak Biosphere Reserve (AABR—22°15′ to 22°58′ N latitudes and 81°25′ to 82°5′ East longitudes) of Central India. Results revealed remarkable differences among Baiga, Gond, Kol, and Oraon ethnic communities and also location (core, buffer and transition) effect on utilization pattern of wild edibles. A sum total of 172 WEPs comprising 60 vegetables, 70 fruits, seeds and nuts, 23 underground tubers and 19 mushrooms were collected, consumed, and surplus were marketed by the communities. On average, the number of wild edibles collected annually by households were in the following quantities: 40–240 kg leafy vegetables, 125–386 kg flowers, 120–250 kg fruits, 12–125 kg legumes, 24–120 kg tubers, 5–35 kg mushrooms. Among ethnic groups, the Baiga primitive community utilized 70–90% followed by Gonds (58–81%), Kols (52–78%), Oraons (43–79%), and other communities (38–68%) in different zones. WEPs have contributed to 5–24% (Rs 3559- 12,710) of household income, which was highest in the core zone and lowest in the transition zone. It was observed that WEPs were complemented the diets rather than being a substitute for staple foods. They supplied only 3.7–8.3% of energy and 1.1–4.9% protein requirements; however, they significantly supplemented ascorbic acid, thiamine, calcium, and iron by 38.1–73%, 13.7–35.4%, 17.2–29.1%, 2.6–13.5%, respectively. Significantly higher quantities of nutrients were supplemented in the core zone compared to other zones. WEPs were currently underutilized (less intake) especially in buffer and transition zones, complementing the staple foods and partially supplementing the essential macro- and micro-nutrients. However, these have the potential to fulfill the dietary needs and ensure balanced nutrition, if consumed in recommended portions and sizes. The paper discusses policy implications that ensure coherence and coordination of local indigenous communities for conservation and sustainable utilization of WEPs of AABR, Central India.

## 1. Introduction

Mainstreaming biodiversity and ecosystem services into food production systems involves strong trade-offs and is critical for balancing livelihoods, culture, habitability, and ecological diversity across heterogeneous landscapes. Biodiversity plays an indispensable role in the maintenance of ecosystem services interlinked to complex socio-economic and biocultural regimes of indigenous communities who have unique values, traditions, beliefs, and lifestyles [1,2,3]. Wild edible plants (WEPs) constitute an important part of household food baskets and form an integral part of traditional ethnic foods across the world, as a looming food crisis warrants exploiting all food resources including WEPs, which are often considered as famine foods significant among aboriginal communities due to their unique sensory acceptability, socio-cultural and spiritual values, recreation and health benefits [4,5,6,7,8]. In recent decades, due to increasing modernization and globalization, the nutritional, ecological, socio-economic, and livelihood benefits of WEPs are well recognized [9,10,11] but are still underrated, neglected, and underutilized in many regions [12]. Undermining the wealth of wild foods impacts the provisioning services of ecosystems and preserving traditional knowledge systems interconnected to indigenous food supply chains that need to be understood in changing lifestyles and environment [13]. However, alarming rates of degradation of productive ecosystems and erosion of cultural diversity across the regions disrupting ecosystem services not only affect the livelihood support of underprivileged indigenous communities but also degenerate the traditional knowledge [14]. Although dietary change, increased investment, policy reforms, biotechnology, and many other proposed solutions hold promise, understanding changing local ethnobotanical knowledge and how communities facilitate ecosystem service delivery can substantially help in biomanipulation and mainstreaming biodiversity conservation in heterogeneous tropical landscapes [3,15].

India has one of the largest concentrations of indigenous people comprising ~104 million people represented by 705 ethnic groups among whom 75 are vulnerable tribal groups. These tribal groups derive multiple products and services from wild habitats [16,17]. The indigenous communities are undisputedly considered to be the weakest marginalized sections of the society facing the brunt of poverty, illiteracy, and backwardness. Despite several ambitious welfare and food security programs having been launched, the expected benefits cannot reach targeted masses due to ineffective implementation and consciousness and, consequently, a large number of indigenous people are subject to food insecure, malnourishment and prone to epidemics not only impeding socio-economic progress but also distressing cultural development [18,19]. Furthermore, WEPs not only contribute to traditional foods but also compliment the culinary value of routine foods and understanding cultural preferences toward different ecosystem services is of great importance as WEPs use has special significance from cultural and nutritional perspectives and prioritizing conservation and/or domestication of vulnerable species. It can also lead to proper planning of rural development through exploiting species having marketing potential and also identifying nutritious species for promoting household income and combating the menace of malnutrition [20].

WEPs have become critical for the sustenance and household income of these groups; moreover, the lower returns from farms necessitate the diversification of income from the sale of WEPs. However, unscientific, concentrated, and over-exploitation of few species degenerating the native diversity of WEPs, while increasing rates of deforestation, epidemics of pests and forest fires in the last few years caused severe genetic erosion disrupting the cultural and traditional food habits resulting in an increase of the incidences of malnutrition and chronic diseases especially among the children and women of indigenous communities. Up until now, very little comprehensive work has been made on the diversity and utilization of WEPs; moreover, studies were concentrated on traditional knowledge for primary health care using herbal drugs in Central India [21,22]. Nevertheless, there has been growing interest in recent years in sustainably exploiting the wild edible resources beyond food and therapeutic uses and intended to understand the local nutrition, dietary diversity, income generation, folklore medicine and safeguard food security through diversification. Indigenous communities meet their diverse demands from surrounding biodiversity and cultural landscape-rich areas of Achanakmaar-Amarkantak Biosphere Reserve (AABR) falling in Central India mainly inhabited by Baiga, Gond, Kol, and Oraon communities who possess significant knowledge about bioresources and their use. Documenting such valuable information is vital for the maintenance of ecosystem services, traditional knowledge, cultural heritage, regulating bio-piracy, biodiversity conservation, development of rural industries, education, employment generation, and ecotourism [23]. This study intended to unveil the contributions on diversity of WEPs and expedite the policy interventions particularly in relation to their quest towards conservation, utilization, and improvement of provisioning ecosystem services among indigenous communities of AABR to explicitly address three specific questions: (i) Whether the ethnic and location differences affect the patterns on divergent use of WEPs? (ii) How much wild plant resources contribute for dietary, nutritional requirements and annual household income of communities? and (iii) What are the strategic interventions needed for conservation and sustainable utilization of WEPs under transforming food systems in AABR?

## 2. Material and Methods

### 2.1. Study Area and Geographic Location of Research Site and Native Communities

**Figure 1:**
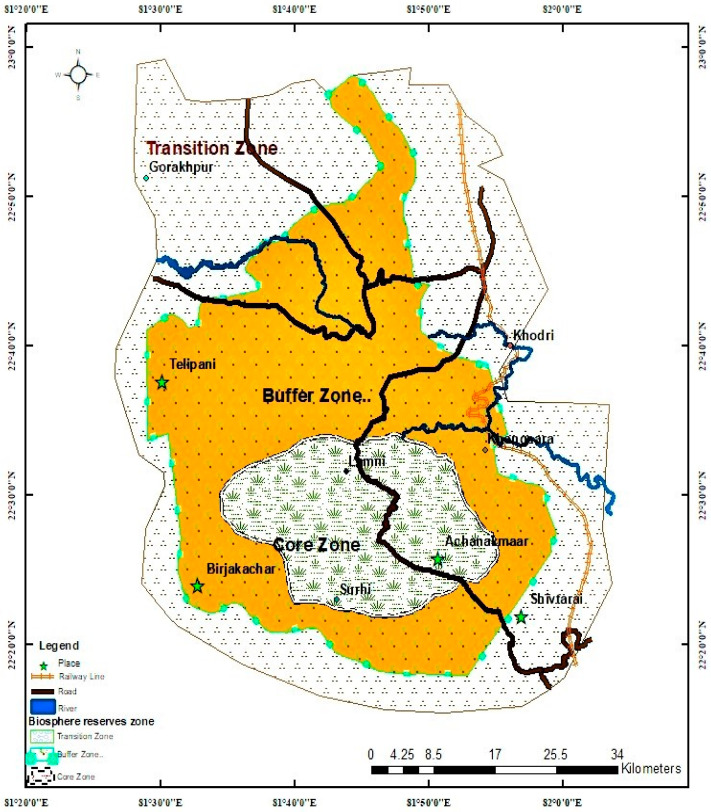
Location of sampling villages in Achanakmaar Amarkantak Biosphere Reserve (AABR) of Central India.

The present investigations were carried out in AABR, the 8th biosphere reserve and declared as a natural heritage site by United Nations Educational, Scientific and Cultural Organization (UNESCO) in the year 2012. It lies between 22°15′ to 22°58′ North latitudes and 810 25′ N to 820 5′ East longitudes and spread over 383,551 ha, of which 68% area covered by Chhattisgarh and 32% by Madhya Pradesh (Figure 1). The biosphere constitutes three zones viz. core (55,155 ha), buffer (195,587.5 ha), and transitional zones (132,809 ha) [24,25]. The entire core zone falls in Chhattisgarh, while buffer and transition zones fall in both Chhattisgarh and Madhya Pradesh (M.P). The biosphere has 411 villages, of which 17 are scattered in the core zone and 394 in other zones of the reserve. The topography of biosphere is mostly undulating slopes with a chain of hills forming Maikal ranges, which connects Satpura and Vindhya hill ranges. The biosphere reserve is covered with typical tropical dry deciduous forests dominated by sal and mixed forests, a plantation of teak, and bamboo [20].

Nearly, 27 communities comprising Baiga, Gond, Dhanwar, Kol, Kanwar, Oraon, Chamar, Sais (Sarthi), Basore, Lonia, Muslim, Sindhi, Brahmin, Rajput, Goswami, Baraith, Kalar, Kumhar, Kewat, Nai, Ahir (Raut), Panika, Sondhiya, Lohar, Maratha, Sonar, and Baniya inhabit core and buffer zones of the biosphere. A sum total of 8168 traditional ethnic tribal inhabitants live in the villages of the core zone, mainly comprising Baiga, Gond, Kol, Pradhan, and Oraons are indigenous communities [20].

### 2.2. Sampling of Villages

The sample was obtained utilizing a stratified two-stage design. The villages were grouped into three strata (Core, Buffer and Transition zones) where each stratum represented a biosphere zone. In the first stage, three representative villages were selected from each of the strata considering demographic features, location, ethnicity, market avenues. Thus a total of nine villages (three from each zone) were selected for household surveys from three zones of the biosphere (Figure 1). A total of 2168 households was present in these villages.

### 2.3. Determination of Sample Size and Sampling of Households

Our study investigates the contribution of wild edible foods (WEF) to the income, energy and nutritional intake of households. A preliminary investigation by the investigators found that the weight of the proportional contribution of WEF in the intake of the households is about 0.5. This proportion is utilized to calculate the minimum sample size (of households) required for the present study. The following expression by Snedecor and Cochran [26] is utilized for sample size determination:

$$\mathrm{Sample}\;\mathrm{size},\;\;\;n=N\times \frac{\frac{Z^{2}\;\times \;p\;\times \;\left(1-p\right)}{e^{2}}}{\left[N-1+\frac{Z^{2}\;\times \;p\;\times \;\left(1-p\right)}{e^{2}}\right]}$$

where, *N* (2168) denotes the population size i.e., total number of households in all the villages, *Z* (1.96) is the critical value of the normal distribution for rejecting a null hypothesis at 5% level of significance, *p* (0.5) is the estimated proportion and *e* (0.05) is the selected margin of error. Using the above expression, the minimum sample size required for the study is 327.

In the second stage, households were sampled from each of the villages selected in the first stage. The probability proportion to size (PPS) method was utilized for the second stage sampling with household size as weight. The criterion was to obtain a sample of 20 or more households from each of the villages depending upon the feasibility as permitted by the terrain. In this process 332 households were selected. In each sample village, households were selected randomly among the communities.

In addition to 332 households, 45 key informants (9 villages × 5 persons representing 20 female and 25 male), 9 focus group members (9 villages × 10 members/group and 30 market vendors (3 markets from each zone × 10 vendors/market) were also questioned to collect information on the list of species, diversity of collection, preparation of recipes, the order of preference and availability of wild edible plants and marketing pattern of WEPs.

Information on qualitative data on collection and utilization of WEPs was obtained through participatory rural appraisal techniques (PRA), direct observation, semi-structured schedule, and key informant interviews. Assistance from local translators and forest department staff was also taken in gathering desired information as most of the female and old respondents were illiterate and conversed in the local tribal dialect. A semi-structured schedule with open-ended questions and a schedule for focus group discussion (FGD) was designed by the stakeholders and a team of experts. The methodology adopted for collecting quantitative information of WEPs are presented in the following section.

### 2.4. Survey on Collection, Consumption and Marketing of Wild Edible Plants (WEPs)

The survey schedule comprises three parts, namely Part-I, Part-II and Part-III. Part-I of the schedule deals with the general socio-economic and demographic profile of the village and households. Part-II of the schedule consists of enquiries pertaining to provisioning services of WEPs, patterns of collection, utilization, sale and marketing pattern of WEPs. It also includes enquiries related to the knowledge of edible plants and their parts. Furthermore, the information on the habitat, the season, typical recipes used in cooking etc. was also collected in Part-II. The specimens of species were collected from the wild with the help of community members and identified. These were matching with records of local flora. The unidentified specimens were photographed and a herbarium was prepared. The unidentified specimens were later identified by expert taxonomists. Part-III of the schedule comprised the questions related to dietary habits, food consumption, and nutrient intake. Parts-I and II of the schedule were carried out in all the households in the sample.

The investigators found that there was no organized marketing with specified market channels for wild edible plants and products. The commodities were traded in local weekly markets popularly known as hat markets. Marketability of wild edibles was studied in three local hat markets of Achanakmaar, Birjhakachar, and Shivtarai villages located in the core, buffer and transition zones respectively. The markets are operated mostly by middlemen and local vendors, who buy raw WEPs along with other commodities in bulk from collectors of indigenous communities at a nominal price on market days and sell them after bundling and washing with a significant margin of profits to local and non-local customers. A marketing survey was conducted in peak hours of the market between 11 a.m. to 3 p.m., where a total of 30 vendors (10 from each market) were interviewed to collect information on the trade of WEPs. The vendors in each of the hat were selected using a systematic sampling plan.

### 2.5. Dietray Consumption and Nutrtion

The Part-III of the schedule was canvassed for a randomly selected subsample of 10 households from among the sample of households selected in the second stage. Food intake data were collected from the selected households representing different indigenous communities in each village by a 24 h recall method repetitively for 3 days each in summer, winter, and rainy seasons. Data were exclusively collected from the women and elderly members as they are involved in cooking and serving food to members of their respective households. The total weight of the prepared dish was recorded and the weight of each raw ingredient was calculated as a percentage of total cooked weight of the dish. The food consumed from conventional and wild edible sources was recorded separately for different food groups. The mean daily per capita intake of foods (average of household members) was recorded and compared with recommended national dietary standards of households (average of working and sedentary men, women and children) as prescribed by the Indian Council of Medical Research (ICMR), Government of India [27,28,29]. The average daily intake of nutrients through various food items was calculated using food composition tables (FCT) for Indian foods and mean data were considered for those species whose values are unavailable [30]. Nutrient values lacking for those foods were replaced by the conversion of data on similar foods in the FCT and all these were compared with the recommended daily allowances [27,28,29].

### 2.6. Statistical Analysis

Descriptive statistics pertaining to the population (total and gender wise), biosphere zone, number of households (total and sampled) and literacy are provided for each of the nine sampled villages. Data on the number and percentage of species and families of WEPs, as well as their life forms and the parts consumed were analyzed using descriptive analysis and frequency calculation techniques [26]. The Chi-square test (χ^2^) was employed for parameters on frequency of collection, utilization and marketing pattern of wild edibles, while a *t*-test for food and nutrition intake was made to compare the significant differences in relation to location and also ethnicity (*p* < 0.05) [26]. The mean consumption (in grams per day by the households) and the per capita consumption of cereals, pulses, vegetables, fruits, tubers, milk and milk products, meat (eggs and fish also), sugar (and jiggery) and oils (and fats) was estimated along with their respective standard deviations. The percent contribution by weight of the WEP in the energy and nutrient intake of the population was also estimated. The statistical analysis was performed in MS Excel and IBM SPSS-23 under PC environment.

## 3. Results

### 3.1. Community Profile and Socio-Economics

The population of studied villages ranged from 429 to 2244, of which 29% to 95% of people represented indigenous communities (Table 1). The number of households varied from 100 to 574, while the literacy rates ranged from 30.6% to 77.1%. Among nine villages, Khodri has the largest population with highest number of households, while Gorakhpur is a small village with lowest population and households. The highest percent (95.3%) of indigenous population to total population was found in Teliapani and lowest (29.1%) in Khodri village. Male–female ratios ranged from 0.97 to 1.26, which was highest in Lamni and lowest in Birjakachar. The number of animal units varied from 10 to 18, while sampled households in the study ranged from 20 to 87 (Table 1). Various plant parts of WEPs viz., leaves, shoots, flowers, pods, fruits, seeds, nuts, roots, tuber, rhizome and roots, were collected from forest lands, wastelands, fringes of agricultural area, water bodies and consumed either raw or in cooked forms as a traditional food in addition to staple cultivated foods.

### 3.2. Utilization of Wild Edible Plant Resources

**Figure 2:**
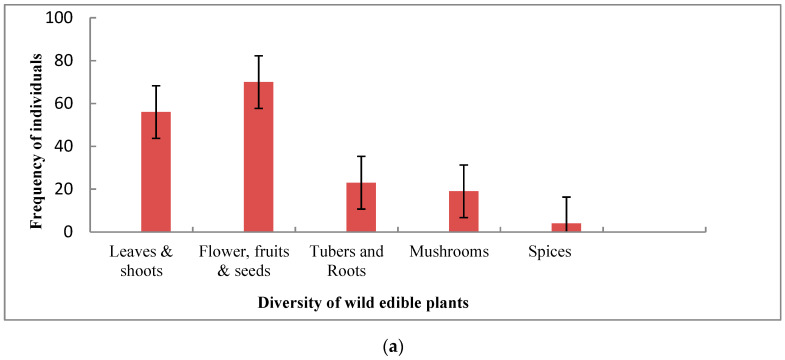

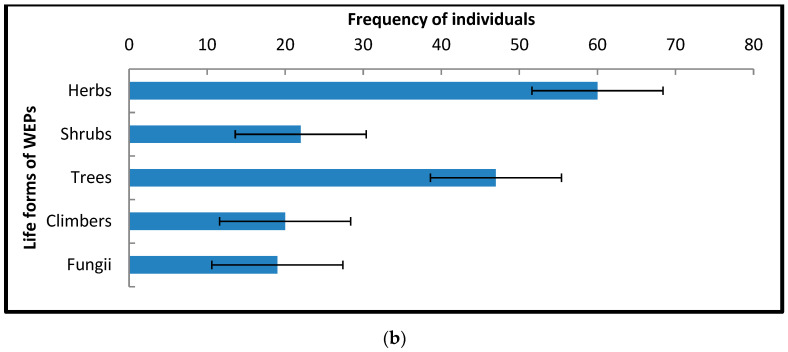
(**a**): Diversity of wild edible plants (WEPs) utilized by indigenous communities of AABR, Central India (N = 332; ±SEm of frequencies); (**b**): Frequency of life forms of WEPs utilized by indigenous communities of AABR, Central India (N = 332; ±SEm of frequencies).

The WEPs resources were essentially categorized into five food categories according to the plant parts utilized by the respondents. A total of 172 WEPs comprising 60 leafy and shoot vegetables, 70 fruits, seeds and nuts, 23 underground tubers, 19 mushrooms. Most of these were consumed by communities and few edibles in demand were locally marketed for securing livelihood and household income (Figure 2a). The wild edible yielding plants (172) were distributed over 5 life forms, of which the frequency of herbs represented 34.9% (60) followed by trees 28.5% (49), shrubs 13.9% (24), climbers 11.6% (20) and fungi 11.1% (19) (Figure 2b).

#### 3.2.1. Wild Leafy Vegetables

All households were using a range of leaves and shoots of WEPs for food, spice, and religious purposes in different seasons of the year supplementing their dietary, cultural, and traditional needs, which could not be completely fulfilled from conventional cultivated species in rainy and winter seasons (Supplement Table S1). A total of 60 species of leafy vegetables corresponding to 35 families were utilized by informants, while in terms of species, the most dominant family was Amaranthaceae (10) followed by Fabaceae (5), Convolvulaceae (5) and Poaceae (3). The edible leaves and shoots were mainly collected from herbs (67%) followed by shrubs (19%), trees (10%) and climbers (5%) and these were harvested from natural habitats including forest lands (38%), agricultural lands (27%), wastelands (25%), wetlands and swampy areas (10%) within a radius of 5–6 km from their villages. The leaves and twigs are usually boiled and cooked as sole vegetables locally called sag baaji but occasionally in combination with pulses, badi, and potatoes. Leaves of different *Amaranthus* species were highly preferred and cooked as baajis are frequently found in traditional diets of all communities; however, frequency was higher in Baigas compared to other groups. The tender shoots of bamboos, locally known as Bans kareel, were highly preferred in local diets and soft shoots often directly eaten after roasting and young shoots cooked as a vegetable and also pickled during the rainy season besides the roasted kareel and shoot sold at 80–100 Rs./- in local markets in different zones of the biosphere. The fresh chutneys were also prepared using mint (*Mentha arvensis* L.) leaves and leaves of amari baji (*Hibiscus sabdariffa* L.) in combination with salt, peppers and spices. Leaves and floral parts of four species viz. *Mentha arvensis* L., *Murraya koenigii* Spreng, *Eryngium foetidum* L. and *Phyllocephalum indicum* K. Kirkman were used as spices to enhance the flavor and taste of traditional dishes by Baigas and Gonds. Based on the culinary value, taste and edible values, the 60 species of leafy vegetables were ranked where 11 and 24 were very high and highly preferred species among the communities, of which 25 species had good market value and locally sold in hat markets. Moreover, 18 species and 6 species were ranked as moderate to least preferred, which were consumed by households in core and buffer zones for their subsistence in times of food scarcity; however, these were seldom found in diets of transition zones, where many cultivated vegetables substitute low valued wild edibles. Fifty percent (50%) of the total wild leafy vegetables (60) were extracted during the rainy season followed by 25% in winter, 17% in summer, and 8% throughout the year, while 58% of these were used variously for religious, timber, non-timber and other edible purposes (Supplementary Table S1).

#### 3.2.2. Wild Edible Flowers, Fruits, Seeds and Nuts

A wide array of flowers, fruits, seeds, and nuts were collected from 70 wild edible species belonging to 34 families; mostly consumed and economically valued species are locally marketed by indigenous communities in AABR (Supplement Table S1). Most of these members belong to the families of Fabaceae (10) followed by Moraceae (6), Cucurbitaceae and Malvaceae (5 each), Rhamnaceae and Rutaceae (4 each), and Anacardiaceae (3). Sixty percent (60%) of the wild edible fruits were collected from trees, while 17% climbers, 16% shrubs, and 7% Herbs. Twenty-five (25) species were collected from the forest habitat, 12 from agricultural lands, 10 from wastelands, and 23 species from homesteads, forest and agricultural lands. Of the total 70 species, 47 yield fruits, 12 flowers, 9 seeds, and 2 nuts as most of them were either directly eaten raw or cooked as a vegetable, pickled, roasted, baked, grilled, smoked, or fermented to make beverages. Unripe fruits, pods and flowers derived from 20 species were cooked as vegetables, the ripe fruits of 43 species were directly eaten, unripe fruits of four species were pickled, and seeds and fruits of three species were roasted and consumed by communities. Among fruit species, Mahua (*Madhuca indica* J.F. Gmel.) is the most preferred and highly valued species, almost all parts of the tree including flowers, fruits and seeds were collected, consumed and marketed economically by communities. Flowers were eaten as raw and also made into chapatis and porridges by mixing with wheat, jowar, and rice flour. This is popular wine usually consumed by all the groups of indigenous communities regularly as the part of cultural heritage. Furthermore, it is also customary to serve liquor to people especially for enjoyment and entertainment during cultural festivals. It is also traditional to serve this drink during ceremonies of marriage, child birth, death, inauguration and opening of new houses, shops etc. The drink is also offered to gods or goddesses as part of the rituals and consumed as a sacred drink. The unripe fruits of Mahua are cooked as vegetables, while ripe ones were collected and seeds were recovered after removing pulp and kernels from Mahua seeds and dried to extract the oil, which is used as edible oil for cooking and occasionally for lighting purposes. Mango is another important fruit yielding species valued for its both ripe and unripe fruits as young unripe fruits were used in making chutney in summer, while the unripe fruits are peeled and dried to make amchoor, which is usually added in vegetable curries and dals to give a sour taste. The unripe mature fruits were boiled with water to prepare ampana as a drink to protect from dehydration during summer. The unripe fruits are also used in making mango pickles, while ripe fruits are directly eaten or juice is prepared and consumed along with rice/wheat chapattis. The leaves and shoots are used in rituals, religious functions and cultural festivals, symbolizing purity, prosperity and spiritual goodness. The mature fruits of Indian goose berry—aonla (*Emblica officinalis* Gaertn.)—were directly eaten, pickled and murabba prepared by mixing with syrups of sugar or jaggery, besides being used in rituals by communities, considered as a sacred tree. Another commercially important species is char (*Buchanania lanzan* Spreng.), where the ripe fruits are directly eaten and kernels extracted from seeds have immense commercial value mixed in sweet dishes and taken along with milk. Munga is another popular species, where young flowers are cooked as vegetables and pods also cooked as vegetables in combination with pulses and soya nuggets. Moreover, ripe fruits harvested from *Diospyros melanoxylon* Roxb. *Tamarindus indica* L., *Aegle marmelos* L. correa, *Annona squamosa* L. etc. were eaten as raw, which have immense potential for commercial exploitation making food and beverages. Of the 70 species, the products of 10 and 12 were very high to highly preferred, which were not only consumed but also sold in local markets due to their commercial value. A total of 25 and 23 of fruits were moderately and least preferred consumed in subsistence but not marketed, while 44 species offer diverse provisioning services, 9 species as religious and cultural, and 7 species provide timber and non-timber products to communities (Table 2).

#### 3.2.3. Underground Stem, Tubers, Rhizomes and Bulbs

Results indicated that 23 species corresponding to 12 families yielding wild edible rhizomes, tubers, bulbs, roots, and underground stems were collected and utilized by indigenous communities (Supplementary Table S1). The highest number of individuals (five) of these species belongs to the Dioscoreaceae family followed by three each in Araceae, Zingiberaceae and two each under families of Asparagaceae, Cyperaceae and Liliaceae. The total documented plant species represented various life forms, 17 species correspond to herbs in their habit and six as climbers, while 13 members provide tubers, four rhizomes, two each suckers, corns and remaining as bulbs. Sixty five percent (65%) of the tubers and rhizomes were collected from forest habitats followed by 22% from wetlands and swamps in winter and summer seasons, most of these were either cooked as vegetables or boiled and even fried as chips. In the rainy season, only seven species provided tubers and rhizomes. Of the total 23 species, 11 were very high to highly preferred tubers collected, consumed and also marketed in hats. The remaining 12 species were usually consumed by households in emergency times of food scarcity. Some important yams and tubers could be safely stored for 2–3 months to serve as alternate foods in times of famine (Supplementary Table S1).

#### 3.2.4. Mushrooms

A total of 19 wild edible mushrooms belonging to eight families were collected and utilized by indigenous communities for household consumption and surplus is sold in local markets (Supplement Table S1). The highest number (six) of mushrooms belongs to the Diplocystaceae family followed by Lyophyllaceae (five), Agaricaceae (three), and Russulaceae (2). The mushrooms usually grow as fruiting bodies of fungus on dead trees of Sal, Bamboo, Paddy straw, and other trees lying on the forest floor found during the rainy season. The mushrooms were consumed in diets after cooking as vegetables by adding salt and spices, or occasionally they were pickled to utilize for longer duration. The mushrooms like *Astraeus asiaticus* Phosri., M.P. Martin and Walting, *Astraeus koreanus* Kreisel, *Astraeus odoratus* M.P. Martin and Whalley, *Russula congoana* Pat., *Termitimyces clypeatus* R. Heim, *Termitimyces heimii* Natarajan, *Termitomyces eurhizus* R. Heim, *Ganoderma lucidum* Karst etc. are commonly found, which were collected and utilized by aboriginal communities. Of the total 19 mushrooms, six were rated as very high and highly preferred, which have good local market value, the surplus quantities were sold at good prices (Supplement Table S1).

#### 3.2.5. Household Consumption, Trading, Income Generation and Potential Threats to WEPs

**Figure 3:**
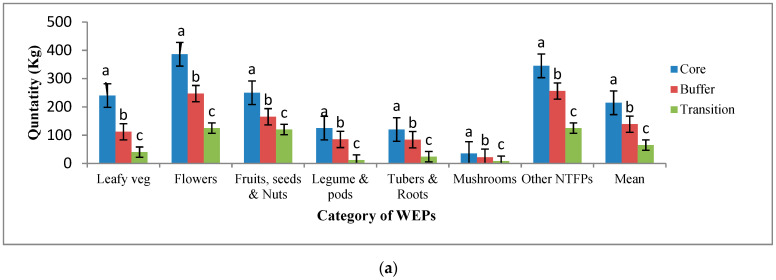

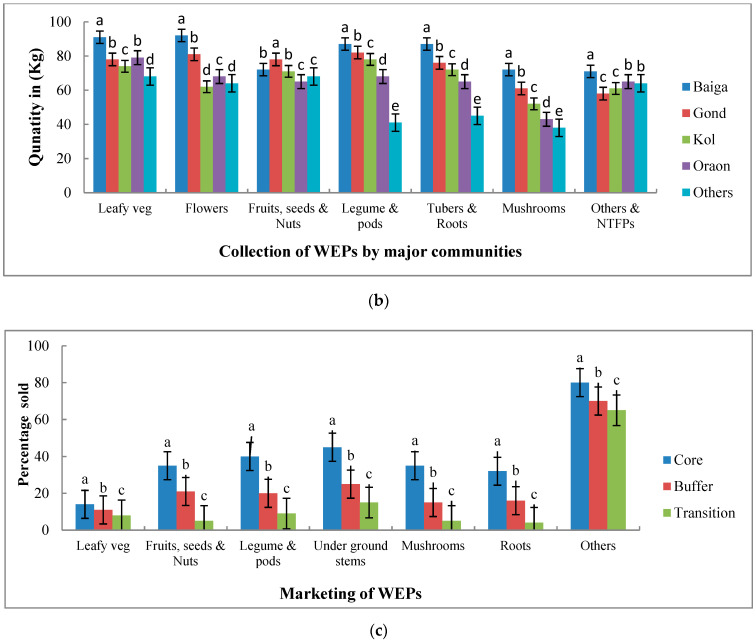
(**a**): Annual quantities of collection of WEPs by households in different zones of AABR, Central India. Different letters denoted on bars for each category of WEPs are statistically different at *p* ≤ 0.05, (N = 332; ±SEm of mean quantities). (**b**): Annual collection of WEPs by major communities of AABR, Central India. Different letters denoted on bars for each category of WEPs are statistically different at *p* ≤ 0.05, (N = 332; ±SEm of mean quantities). (**c**): Marketing (Yearly) of WEPs collected by households in different zones of AABR, Central India. Different letters denoted on bars for each category of WEPs are statistically different at *p* ≤ 0.05, (N = 332; ±SEm of percentages).

It was also observed that a significantly large number of WEPs and their parts were gathered and consumed in households by indigenous people living in core and buffer zones of AABR (Figure 3a).

**Figure 4:**
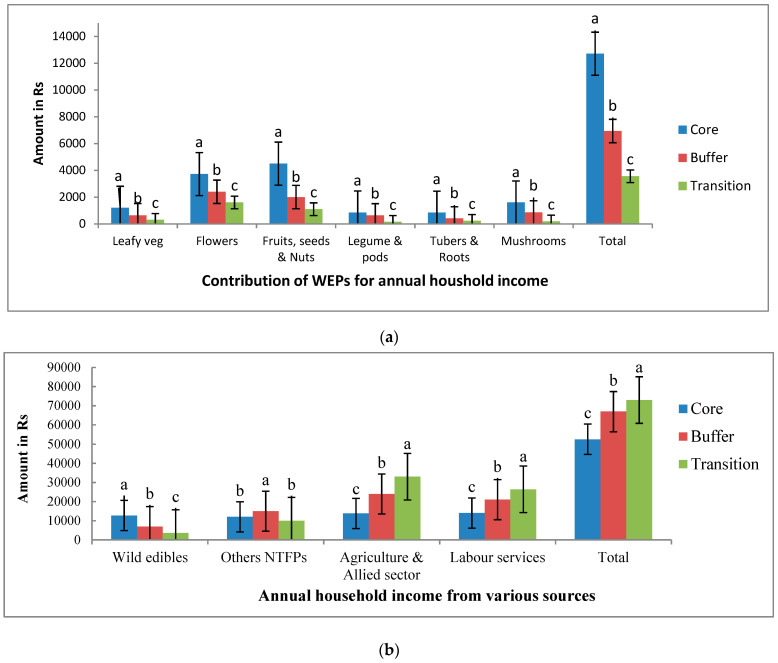

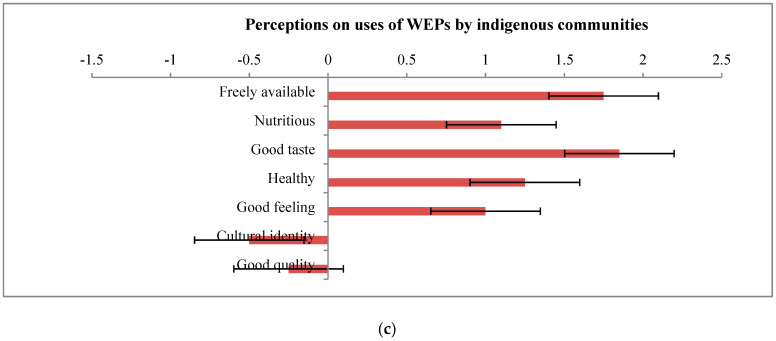
(**a**): Contribution of WEPs for household income of indigenous communities of AABR, Central India, Different letters denoted on bars for each category of WEPs are statistically different at *p* ≤ 0.05 (Note: Indian Rs 73/- equals to 1 US D) (N = 332 ±SEm of amount in Indian Rs). (**b**): Household income from major sources of indigenous communities of AABR, Central India, Different letters denoted on bars for each category of WEPs are statistically different at *p* ≤ 0.05 (Note: Indian Rs 73/- equals to 1 US D) (N = 332; ±SEm of amount in Indian Rs). (**c**): Perceptions on use of WEPs indigenous communities of AABR, Central India on a 5 point Likert scale (N = 332; ±SEm of scores).

The collection of wild edible plants by households was in the order: flowers > fruits > leaves > legumes and pods > other NTFP (non-timber forest product) (Sal gum—*Shorea robusta* Roth. Mahul leaves—*Bauhinia vahlii* Wight and Arontt, Karaya gum—*Sterculia urens* Roxb., Tendu leaves—*Diospyros melanoxylon* Roxb.), honey, gums, dyes > tubers > mushrooms (Figure 3a). On an average, the number of wild edibles collected annually by households in three zones was in the following quantities: 40–240 kg leafy vegetables, 125–386 kg flowers, 120–250 kg fruits, 12–125 kg legumes, 24–120 kg tubers, 5–35 kg mushrooms, and 125 to 345 kg other miscellaneous NTFPs (Figure 3a). Among ethnic groups, the wild edibles are popular among the Baiga primitive community as they utilize about 70–90% followed by Gonds (58–81%), Kols (52–78%), Oraons (43–79%), and other communities (38–68%) in households (Figure 3b). After meeting the dietary needs, the surplus amounts of wild edibles collected by households in demand were sold in weekly hat markets spread in core and buffer zones, while daily vegetable markets in the transition area of biosphere. It was estimated that 8–14% of leafy vegetables, 5–35% of fruits, 9–40% of legume and pods, 15–45% of tubers, 5–35% mushrooms, 65–80% other NTFPs were collected and sold by households (Figure 3c). Utilization and marketing of wild edibles were highest among people of the core zone followed by buffer and transition zones (Figure 3c). The average annual household income generated by the sale of WEPs was highest among indigenous people of the core zone followed by buffer and transition zones. The income contributed from WEPs ranged from Indian Rs 3559 to Rs 12,710 to total annual household income across three zones (Figure 4a).

Among the annual household income generated from the trading of WEPs, the sale of flowers and fruits alone contributed 60–70% (Rs 2700- Rs 8220/- 1 US$ = Rs 73) of household income across three zones of biosphere followed by mushrooms (5.1–12.6%; Rs 180–1600/-)), leafy vegetables (8.4–9.4%; Rs 300–1200/-) and tubers (5.9–6.6%; Rs Rs 225–840), and legumes and pods (4.3–6.7%; Rs 154–850) (Figure 4a). The flowers of Mahua (*Madhuca indica* J.F. Gmel., fruits of tamarind (*Tamarindus indica* L.), aonla (*Emblica officinalis* Gaertn.), sitaphal (*Annona squamosa* L.), jamun (*Syzygium cumini* (L.) Skeels), mangoes (*Mangifera indica* L.), seeds of char (*Buchanania lanzan* Spreng) etc. were major contributors for household income, whereas the very highly preferred leafy vegetables such *Amaranthus* sp., Mushrooms (Raja bodo, sarai, pyra putto) and tubers wild yams, bamboo shoots possessing good demand in local markets had also significantly contributed for household income. The price list of 71 WEP species commonly sold in local hat markets are summarized in Supplementary (Table S2). Both female and male members were involved in harvesting and collection of WEPs in almost equal proportions in the core zone, whereas they were male in buffer and transition areas. The processing and storage were mainly done by women, while marketing of commodities of WEPs by men and women.

The communities derive household income from diverse sources including agriculture and allied sectors, labour and trade of wild edibles, and other NTFPs. The household annual income ranged from different sources ranged from 12,000 ± 1240 Rs. to 14,000 ± 1380 Rs., 7000 ± 840 Rs. to 24,000 ± 2460 Rs. and 3559 ± 840 Rs. to 26,400 ± 2840 in core, buffer, and transition zones, respectively (Figure 4b). It was highest (72,959 ± 5100 Rs.) in transition zone followed by buffer (66,937 ± 4840 Rs.) and core zones (52,510 ± 4050 Rs.). The wild edibles contributed for 5–24% of household income, which was highest in the core zone and lowest in the transition zone, while these accounted for only 11% and 5% of total income in buffer and transition zones, respectively (Figure 4b). The key perceptions on use of wild edibles by indigenous communities were scored on a 5-point Likert scale and the results are presented in Figure 4c. The seven attitudes were ranked on acceptance of WEPs to integrate in diets by indigenous communities. The good taste and free availability of WEPs are the two primary reasons, which achieved the maximum scores of 1.9 and 1.8, while the other three perceptions of the nutritious, healthy and good feeling nature have been scored between 1–1.2 ranked as secondary motives (scored between 1–1.25). In contrast, the indigenous communities did not consider the cultural and religious attributes were that important compared to other reasons mentioned above and thus gave low scoring to these factors by informants (Figure 4c). The potential factors of threat on availability of WEP were scored on a 5-point Likert scale (threat levels, 5—Very high, 4—High, 3—Moderate, 2—Least threat, 1—No threat) and these scores summed across zones to obtain total score out of a maximum 15, the factor with highest score is ranked in Roman numerals (I–X) (Table 2).

The level of threats of wild edible plants is quite variable in different zones of AABR. Expansion of agriculture into native forests is rated as number 1 by communities and observed as a major threat to future supplies of WEPs in AABR. The other factors like overharvesting and faulty harvest methods, changing demands and marketing, overgrazing and over harvesting of timber and firewood are also recognized as potential threats. Some of the timber and firewood-yielding species like sal (*Shorea robusta* Roth.), tendu (*Diospyros melanoxylon* Roxb.) aonla (*Emblica officinalis* Gaertn.), char (*Buchanania lanzan* Spreng) etc. also provide WEPs. On the other hand, the construction of minor dams, protected mining and expansion of roads has been recognized as not a major threat by communities (Table 2).

### 3.3. Consumption Pattern of Diverse Food Groups and Intake Pattern of Nutrients

**Figure 5:**
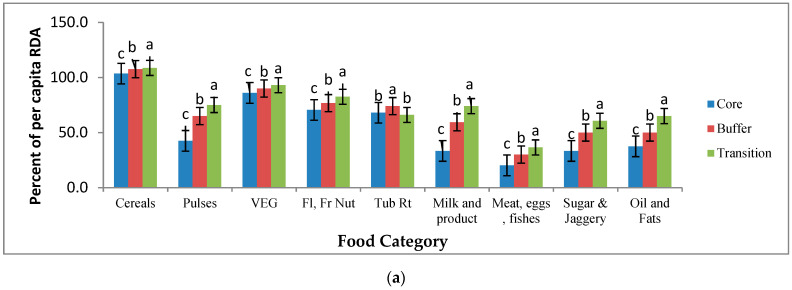

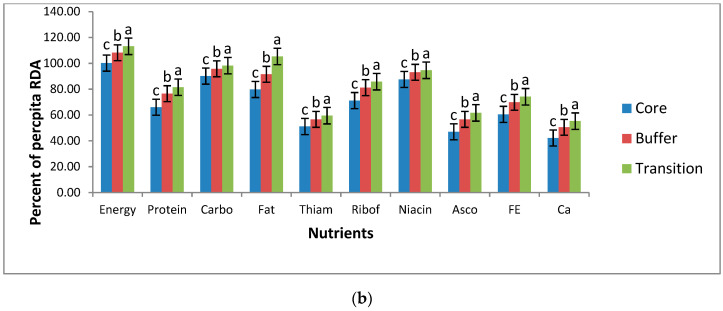
(**a**): Per capita intake of food categories as percent of recommended dietary allowances (RDA) by indigenous communities in AABR, Central India, Different letters denoted on bars for each category of foods are statistically different at *p* ≤ 0.05 (N = 332; ±SEm of percentages). (**b**): Per capita intake of nutrients as percent of RDA by indigenous communities in AABR, Central India, Different letters denoted on bars for each category of nutrients are statistically different at *p* ≤ 0.05 (N = 332; ±SEm of percentages). Note: Carbo—carbohydrates, Thiam—thiamne, Ribof—riboflavin, Asco—ascorobic acid, Fe—iron, Ca—calcium.

The per capita food consumption (g/day) pattern revealed that, except for cereals, all other food items were consumed in lower quantities in comparison to recommended dietary allowances (RDA) prescribed by the Indian Council of Medical Research (ICMR), Government of India (Table 3). The per capita intake (average of working and sedentary men, women and children of household) of different food groups at household level was higher in transition followed by buffer and core zones. Meat, eggs, fishes, animal and milk products, pulses, sugar and jaggery and fats were consumed in substantially lower quantities than vegetables, fruits and tubers at household level in all three zones (Figure 5a). The consumption of meat, eggs, fish, pulses, sugars, oil and fats was less than 50% of recommended dietary allowances in the core zone (Figure 5a). On the other hand, the intake of vegetables, fruits, tubers, milk and milk products ranged between 70–80% of RDA in transition areas. Overall, the intake of food items among indigenous communities of AABR as compared RDA at house hold level (Average of all family members) was lower by 39.2%, 10.3%, 23.7%, 30.6%, 44.4%, 71%, 52% and 49.1% for pulses, vegetables, fruits, tubers, milk, meat, sugar and fats, respectively (Figure 5a).

The per capita intake of macro- and micro-nutrients by indigenous communities was lower than recommended quantities (Table 4). It was comparatively higher in the transition zone followed by buffer and core zones. Except for the ascorbic acid, fats, and iron, all other nutrients were inadequate and below the RDA even in the transition zone. Communities in both core and buffer zones were meeting neither protein requirements nor micronutrients through their dietary habits (Figure 5b). In the transition zone, the nutrient intake in households was 10–25% higher than the core zone. Among all macronutrients, the consumption of proteins was significantly lower (55–67%) than fats and carbohydrates as their diets had limited plant and animal protein. Similarly, the intake of ascorbic acid, calcium, thiamine and iron were lower compared to niacin and riboflavin (Figure 5b).

Wild edibles especially leafy vegetables, legume pods, other vegetables, fruits, flowers, mushrooms, roots and tubers had contributed significantly (*p* ≤ 0.05) to supplementing micro- and macro-nutrients (Table 5). The WEPs have contributed <10% for energy and protein requirements but supplied essential micro nutrients. The wild edibles contributed on an average of 38.1–73%, 13.7–35.4%, 17.2–29.1%, 2.6–13.5% ascorbic acid, thiamine, calcium, and iron, respectively. Significantly higher quantities of nutrients were supplemented in the core zone. On average, WEPs contributed 6.2% for energy, 3.3% protein, 4.6 % carbohydrates, 7.4% fat, 26% thiamine, 7.4% riboflavin, 3.3% niacin, 56.1% ascorobic acid, 3.5% iron and 24.3% calcium across the zones.

## 4. Discussion

Disruption in ecosystem services from dwindling WEPs resources that contribute to social, cultural, environmental and economic development poses a serious threat to the food and nutritional security of indigenous people. Several studies suggested developing resilient food production and supply systems and promote the sustainable use of traditional foods, which should be seriously considered while formulating policies, practices, technologies, and strategies that lead to the conservation and sustainable development of WEPs. These resources provide valuable provisioning services securing food, timber, firewood, medicine, other NTFPs and ensuring sustained income and supplementing food in famine times and nutrition for underprivileged indigenous communities in India [31,32,33]. Our study documented a range of wild edible plant sources yielding leafy vegetables, shoots, flowers, fruits, tubers, roots, legume pods, mushrooms, etc. were diversely utilized by ethnic communities in the AABR region of Central India, which are congruent with reports of studies conducted elsewhere [34,35,36]. Arinathan et al. [36] reported a total of 171 wild edible plants representing 67 families were extensively used by the Pallayaris tribe in the Western Ghats region of Tamilnadu, southern India. A wide range of plant parts such as rhizomes, corns, tubers, bulbils, and roots of 19 species, stem pith and apical meristems of 12 species, leaves of 54 species, flowers of 10 species, unripe fruits 41 species, ripe fruits of 64 species and seeds and kernels of 45 species were consumed raw or cooked as a vegetable. Similarly, Sandriyal and Sandriyal [34] recorded 192 species of wild edible plants were consumed and 47 were sold in local markets by indigenous communities in the Sikkim Himalayan region of India. Ghorbani et al. [37] recorded 173 species representing 64 families and one species of lichen (Ramalina sp.) were used as WEPs in Yunnan, southwest China, while, Kala [38] documented the use of only 73 WEPs species by indigenous and other communities in Chhattisgarh, India. All these studies show that the number and frequency of WEPs species used vary according to geographic location, abundance, traditional knowledge, shortage in conventional foods, and the socio-cultural and economic conditions of communities.

Among the communities, the Baigas collect and consume a large number of wild edible resources in different food groups compared to Gonds, Kols, Oraons, and other communities in AABR. The ethnic differences in collection and utilization of WEPs were also widely reported across the world, which was perceived as diverse cultural and environmental settings rather than economic conditions [11,32,34]. Termote et al. [39], while comparing the traditional knowledge of WEPs among three ethnic communities in the Tshopo district of DR Congo, reported that utilization and traditional wisdom of WEPs were culturally highly diverse between ethnic groups. Our results further corroborated with fact that Baigas utilized 30% of wild edible species for food and fruits, in the Baigacheck area in Dindori district of MP in central India [40]. A high frequency of WEPs consumption by the Baiga community in the core region could be attributed to their rich traditional knowledge, religious and customary needs, moreover, they were well acquainted with how efficiently WEPs could be utilized for food, and income generation in their socio-cultural milieu. The ethnicity and locational differences in utilization of WEPs were widely recognized, which has been mainly attributed to the socio-cultural background rather than diversity and accessibility as utilization patterns of WEPs were driven by culture, traditions, knowledge and biogeographical factors [41].

### 4.1. Household Income and Contribution of Wild Edibles

The wild edible plants were found to be a significant source of livelihoods and income to households of indigenous communities especially in core and buffer zones of AABR as products derived from more than 70 were seasonally sold in the weekly local hat markets and in return the communities buy groceries and essential goods for basic household needs. The differences in household consumption and marketing pattern of WEPs due to different geographic locations were consistent with reports of earlier workers [42,43,44]. Lepcha et al. ([44] observed significant contribution of NTFPs to household monthly income (1–70%) among indigenous communities of Jaldapara National Park, India. Forty-three (43) species provided leaves, shoots, fruits, nuts, seeds, twigs, mushrooms, and fish that were sold in local markets, while Nudeck et al. [45] remarkably reported that sale of WEPs contributed between 1–100% to household income in Shorobe village in northern Botswana.

The primary collectors in the study area are indigenous communities who sell their products to middlemen at nominal prices. Ahirwar et al. [46] while studying on marketing of the wild edible fruits reported that primary collectors realized only 16%, while the middlemen obtained 67% share of the market price. WEP marketing patterns revealed that it is an unorganized sector as there is neither any specific market channel nor any institutional mechanism. This calls for institutional intervention and arrangement for assuring the minimum support prices for many important products; of late only 15–25 products of WEPs were included in the support price list and only 4–5 products (leaves of *Diospyros melanoxylon* Roxb. and *Bauhinia vahlii* Wight and Arontt, gum of *Sterculia urens* Roxb., seeds of *Shorea robusta* and lac) were nationalized with a proper institutional arrangement of procurement by the state forest department. Numerous researchers have argued for the need to improve market facilities and support prices for smooth trading of a range of WEPs to avert the ongoing exploitation of indigenous communities by middlemen [34,43].

The study also revealed that wild edibles contributed to 5–24% (Rs 3559 to Rs 12,710) for annual household income (Rs 52,510 to Rs 72,959) across three zones in AABR, Central India. Although the contribution of WEPs for income was significant but quite low, this could be increased through primary processing and value addition, which will not only augment economic returns but also enhance the shelf life of perishable commodities. A number of fruits and vegetables that were sold as raw in the markets at low prices could be easily processed in the form of pickles, chutney, jam, jelly, squash dried fruit, dried powder, dried seed, marmalade, etc. with minimum costs and ordinary skills. At least 20–30% of locally popular wild edible fruits such as mango (*Mangifera indica* L.), ber (*Ziziphus jujuba* Mill), bael (*Aegle marmelos* L. correa) lasorda (*Cordia myxa* L.), aonla (*Emblica officinalis* Gaertn), seethaphal (*Annona squamosa* L.), tendu (*Diospyros melanoxylon* Roxb.). wood apple *(Limonia acidissima* L.), char (*Buchanania lanzan* Spreng), karonda (*Carissa carandas* L.), imli (*Tamarindus indica* L., munga (*Moringa oleifera* Lam.) etc. shoots of bamboos, yams (*Dioscoreas*) thikhur (*Curucuma angustifolia* Roxb.), spices, mushrooms, etc have strong potential for processing and value addition.

### 4.2. Food and Nutrition Intake

The food consumption (g/day) pattern revealed that diets of indigenous communities of AABR were purely cereal based, while the intake of pulses, vegetables, fruits, tubers, milk, meat, sugar, and fats were rather less than the recommended dietary allowances (RDA) prescribed by ICMR. The non-availability of foodstuffs in core and buffer zones or prevailing access to the public distribution system are the main causes of inadequate consumption below the recommended dietary allowances compared to transition zone. The scientific agriculture practices, marketing facilities, along with effective public distribution and better socio-economic conditions propmted the use of adequate quantities of conventional foods resulting in lesser dependency on WEPs in the transition zone. Moreover, people are changing their indigenous lifestyles and accepting the alternative sources of foods, gradually shifting from tradtional food habits. The divergent variations in dietary consumption and low intake of food-stuff were not uncommon, which had also been reported previously among indigenous communities in India [47,48,49].

Turning our attention to the pattern of consumption of foods and nutrients, it was evident that intake of macro and micro-nutrients by indigenous communities were less than desired allowances, as a consequence of lower consumption of recommended food groups, especially those that meet the requirement of proteins, vitamins, and mineral nutrients are lacking in their diets. Although WEPs especially leafy vegetables, legume pods, other vegetables, fruits, flowers, mushrooms, roots and tubers significantly supplemented the household food requirements in the core and buffer zone, these were still inadequate. Studies conducted in the past suggested that WEPs were important in native food systems and contribute to food and nutrition security of the poor [5,6,8,50].

Our study showed that the contribution of wild foods to total food consumption was rather low among indigenous communities of AABR.This has resulted as a consequence of intake of lower quantities of WEPs thus only meeting the partial requirements of micronutrients leaving a big deficit, which could be fulfilled by increasing their quantities in traditional diets in core and buffer zones. There is vast potential to exploit nutrient-rich underutilized WEPs in large quantities than the present level of consumption in AABR. Jain and Tiwari [51] emphasized the leaves of *Oxalis corniculata* and *Cassia obtusifolia* are nutritionally rich in crude protein (20.2–22.3%), lipids (23–23.7%), and total sugars (4–12.1%). Nutrient-rich neglected WEPs could help transform food systems, if science and policy are well connected with better coordination among the diverse stakeholders working on conservation and sustainable utilization [11,23,52].

The present study demonstrated the significant role and contribution of a wide array of WEPs (172) for meeting dietary needs and household income (5–12%) of indigenous communities, however, these have been largely underscored and have not received enough attention in government programs. WEPs and their products need to be promoted and incorporated into various nutritional and food supply schemes of the government comprehensively, especially under integrated women and child development programs, mid-day meals of schoolchildren, and a public distribution system for meeting diverse needs leading to the holistic development of indigenous communities. The National Food Security Act of Government of India, 2013 has included highly nutritious and climate resilient minor millets in the public distribution systems to benefit millions of schoolchildren and the wider population, and in a similar way the potential nutritious wild foods could be linked to these programme and fostered in habitats that are consumed along with staple foods in addressing the problems of undernourishment. There is an immense potential for bioprospecting of genetic resources of WEPs and traditional knowledge for developing herbal medicines, crop protection, cosmetics, horticulture, agricultural seeds, environmental monitoring, however the ethical consideration in fair and equitable sharing benefits to the indigenous communities are often neglected, undermined and that often create conflicts among stakeholders [53,54,55].

The collection of wild edibles viz. Buchanania lanzan Spreng., Emblica officinalis Gaertn., Terminalias, Syzygium cumini (L) Skeels, Ziziphus jujuba Mill., Cordia dichotoma G. Forst., Asparagus racemosus Willd., Dioscorea bulbifera L., Boswellia serrata Roxb., etc. were done by heavy lopping of branches, plucking twigs, removal of immature tubers, and sometimes even scarifying trees by felling. The unscientific and over-harvesting have been widely recognized as potential threats for eroding the diversity of WEPs along with other trade in AABR thus affecting the food and nutritional security of communities, which coloborate with the finding of the earlier studies [3,38,42,52]. The contextual problems draw attention to the need for a strategic policy that includes a comprehensive conservation plan of WEPs for safeguarding and ensuring a sustainable supply of ecosystem services by involving local communities with social cohesion and participation embracing the cultural values and traditions to protect eroding bioculture from rapidly diffusing urban culture [51,52]. Our results can serve as baseline data for evolving suitable adaptive and coping strategies under changing socio-ecological conditions to strengthen the ecosystem services and traditional knowledge of WEP species and bioculture, which is an important component of the first and second Sustainable Development Goals (SDGs).

The past few decades has witnessed rapid degradation of native forests of AABR, Central India [42], lead to erosion of a large number of indigenous plants including WEPs which has simulated an interest in conserving the eroding resources to ensure sustainable supplies of WEPs to address the looming crisis of food scarcity, malnutrition and livelihood security among indigenous communities. The present study revealed that the major causes behind the loss of diversity of WEPs are encroachment and expansion of agriculture inside forest areas, over–harvesting, and faulty harvesting of wood and non-wood products due to increased biotic pressure. Nevertheless, the contribution of WEPs to food and nutrition are well known but very little attention is given in conservation in the AABR region [56]. A number of studies have demonstrated the decreasing availability of WEPs in natural ecosystems across the country and the loss of WEPs was due to habitat degradation, over exploitation, as well as changes in food habits [7,57]. The use of fertilizers, herbicides pesticides, mechanization and unsustainable harvest methods further damaging the regeneration of valuable species. A coordinated concerted effort is needed from all sectors to implement sustainable management and conservation of WEPs, which are currently neglected in AABR. The involvement and participation of local communities Baigas, Gonds and Prdhans along with state forest department, educational institutes, environmental bodies, non-governmental organisations (NGOs) and Gram panchayats will craft more holistic and culturally appropriate strategies for utilization and management of WEPs in the AABR [42]. The local people are real custodians and motivated towards sustainable management as they are both the guardians and users of the WEP resources and have the greatest knowledge about them. The conservation and utilization of the indigenous knowledge of useful plants can help in the improvement of livelihoods of indigenous communities which needs to be boosted to preserve the bio-cultural regimes which are deteriorated by the influx of alternate foods gradually replacing the value of traditional foods. WEPs play a significant role in meeting daily food requirements and an important mechanism of survival for people living in the core and buffer zones of AABR and interest will be created among younger generations through awareness programmes and local fairs. *In-situ* conservation of rare, endangered and threatened species like aonla (*Emblica officinalis* Gaertn.), char *(Buchanania lanzan* Spreng.), lasooda (*Cordia myxa* L.), mahua (*Madhuca indica* J.F. Gmel.), tendu (*Diospyros melanoxylon* Roxb.) karaya (*Sterculia urens* Roxb.), salai (*Boswellia serrata* Roxb.) etc. needs to be promoted by planting species communities under various programmes. The payment to communities rendering services of conservation and restoration of degraded forests shall be ensured through REDD+ (Reducing Emissions from Deforestation and Forest Degradation) and JFPM (Joint Forest Planning and Management) programmes. Domestication of WEPs in buffer and transition zones needs to be encouraged to ensure continued availability; thus, it would provide multiple benefits, promote their livelihoods and income [58]. The encroachments and expansion of agriculture into forest lands could be minimized by granting land tenure settlement to communities as per the Forest Rights Act (2006). Our results can serve as baseline data on WEPs and identifying critical gaps useful while formuating policies of food and nutrtional security among communities, which is an important component of the first and second goals of SDG. The study has been conducted to holistically address the food and nutritional issues in relation to the use of WEPs; however, further investigations are needed in more detail on the food and nutrtional aspects with reference to gender, age groups and different sites for a better understanding and sustainable utilization of WEPs in AABR.

## 5. Conclusions

A total of 172 WEPs providing leafy vegetables, fruits, seeds, nuts, roots, shoots, rhizomes, tubers, mushrooms, etc. were utilized as a source of foods by indigenous communities across different zones of AABR, Central India. The study showed that people living in core and buffer areas of AABR mostly rely on wild edibles supplenenting the requirements of food and nutrition. Baiga, an underprivileged primitive community, possess more traditional knowledge on diverse uses of WEPs, thus exploiting comparatively a higher number of wild edibles for meeting household food and nutrtional needs. The study revealed that livelihoods and economy are intricately linked to traditions and values that are deeply rooted in the culture of Baigas, Gonds, Pradhans, Oraons, Kols of the AABR, while WEPs contributed significantly to the household incomes of these communities; however, the income levels were much lower in transition and buffer zones. The middlemen were key players, exploiting poor communities by procuring the valuable WEPs at nominal prices; therefore it is suggested to develop appropriate mechanisms and evolve institutional arrangements for marketing of WEPs at assured support prices at least for a range of popular commodities so that legitimate benefits could be realized. The processing and value addition of wild flowers, fruits, nuts, mushrooms, etc. needs to be promoted through cooperatives or self-help groups of communities that could not only increase quality, shelf life but also ensure higher income than current levels. The study also indicated that diets of indigenous communities were cereal-based, while the consumption of other commodities was in inadequate quantities, whereas WEPs were supplementing food, essential macro- and micro-nutrients. Nonetheless, the present levels of intake appear to be inadequate but they have potential to meet the total dietary needs if taken in recommended portions and sizes, which will not only add dietary diversity but also overcome the nutrient deficiencies especially in core and buffer zones, where both indigenous populations and WEPs are largely concentrated. The unscientific approach and overexploitation leading to degeneration of valuable wild edible fruit-yielding species like aonla, char, mahua, tendu, bohar etc. affect their frequency and abundance, therefore suitable management interventions were suggested to conserve the vulnerable species by involving the indigenous communities. Moreover, the illegal expansion of agriculture into forested landscapes also eroding the diversity of WEPs in AABR. The Forest Rights Act (2006) will provide a solution to permanent land tenure to communities and discourgae the evil practices affecting the abuandance and diversity of WEPs. In situ and ex situ conservation measures could help in regeneration and preservation of endangered and threatened species, which can ensure financial incentives to local stakeholder communities through JFPM, REDD^+^ and MGNREGA (Mahatma Gandhi National Rural Employment Gurantee Act, 2005) programmes. The management and promotion of WEPs systematically and sustainably would not only improve food and nutrtional security but also build socio-economic resilience and create novel opportunities for bioprospecting of potential resources. However, sensitive policies and programmes should be evolved to ensure fair and equitable sharing of benefits between communities of AABR and users of biological resources and indigenous knowledge.

## Figures and Tables

**Table 1:** Socio-demographic characters of sample villages in Achanakmaar Amarkantak Biosphere Reserve.

Sl. No.	Village	Latitude	Longitude	Zone	House Holds	Total Population	M	F	L (%)	IC (%)	AU	Sample Households
1	Achanakmaar	Lormi	Mungeli	C	130	535	283	252	64.18	36.1	15	20
2	Surhi	Lormi	Mungeli	C	205	802	400	402	55.60	79.1	16	31
3	Lamni	Lormi	Mungeli	C	147	634	354	280	57.56	78.1	18	22
4	Birjakachar	Lormi	Mungeli	B	286	979	481	498	30.60	70.5	18	43
5	Khongsara	Kota	Bilaspur	B	266	1052	526	527	57.44	75.2	12	40
6	Telipani	Pandariya	Kabeerdham	B	135	607	301	306	59.00	95.3	15	20
7	Gorakhpur	Dindori	Dindori	T	100	429	224	205	37.32	86.1	10	20
8	Khodri	Gurella	Pendra Gurella Marwahi	T	574	2244	1127	1117	77.14	29.1	11	87
9	Shivtarai	Kota	Bilaspur	T	325	1297	641	656	76.19	82.1	10	49
Total					2168							332

Note: M—Male; F—Female; L—Literacy (%); IC—Indigenous communities; AU—Animal unit; C—Core zone; B—Buffer zone; Transition zone.

**Table 2:** Ranking of threats to WEPs in AABR, Central India (scored on 1–5 point Likert scale N = 332, Arabic letter denotes score, Roman letters indicate ranks).

S. No.	Factors	Core	Buffer	Transition	Total Score	Rank
1	Overgrazing	2	2	4	9	IV
2	Overharvesting and Faulty harvest methods	2	4	5	11	II
3	Encroachment for Agriculture	3	4	5	12	I
4	Uncontrolled forest fires	1	1	4	6	VII
5	Frequent droughts	2	2	3	7	VI
6	Protected mining activities	1	1	2	4	IX
7	Firewood and Timber	2	3	3	8	V
8	Expansion of roads and communication	1	2	2	5	VIII
9	Minor dam construction	1	1	1	3	X
10	Changing demands and marketing	4	3	3	10	III

**Table 3:** Per capita food groups intake (consumption- g/day) pattern of Indigenous communities in AABR, Central India.

Zone and RDA	Food Group								
	Cereals	Pulses	Veggies	Fruit	Tubers	Milk and Product	Meat, Eggs and Fishes	Sugar and Jaggery	Oil and Fats
Core	476 ± 3.6 b	17 ± 1.5 b	86 ± 2.5 b	70.5 ± 2.8 c	34.0 ± 3.2 b	50.0 ± 2.5 c	8.2 ± 1.1 c	13.0 ± 1.9 b	20.0 ± 1.5 c
Buffer	495 ± 3.8 a	26 ± 2.1 a	90 ± 3.6 a	76.0 ± 2.5 b	37.0 ± 3.4 a	89.0 ± 1.6 b	12.0 ± 1.8 b	15.0 ± 1.8 ab	22.0 ± 1.8 b
Transition	500 ± 4.2 a	30 ± 2.5 a	93 ± 2.9 a	82.5 ± 3.6 a	33.0 ± 2.8 b	111.0 ± 3.5 a	14.6 ± 2.2 a	18.2 ± 2.4 a	26.0 ± 1.9 a
Average	490.3	24.3	89.7	76.3	34.7	83.3	11.6	15.4	22.7
RDA	350	80	250	100	150	300	35	25	25

Note: Values in the column followed by different letter are statistically significant at *p* ≤ 0.05 level (N = 90, 30/zone ± SEm of quantities).

**Table 4:** Per capita nutrient intake (per day) pattern of Indigenous communities in AABR, Central India.

Zone and RDA	Nutrients									
	Energy (K Cal)	P (g)	C (g)	F (g)	T (mg)	R (mg)	N (mg)	A (mg)	Fe (mg)	Ca (mg)
Core	2203.9 ± 54.3 b	33.0 ± 2.3 b	360.4 ± 14.2 c	30.3 ± 2.1 c	1.0 ± 0.1 b	1.1 ± 0.2 b	13.1 ± 1.1 b	32.9 ± 2.4 c	13.9 ± 1.4 b	421.9 ± 24.5 c
Buffer	2379.7 ± 68.9 ab	38.3 ± 2.8 a	382.9 ± 213 b	34.8 ± 2.3 b	1.1 ± 0.2 a	1.3 ± 0.1 b	14.0 ± 1.5 a	39.6 ± 2.8 b	16.1 ± 1.2 a	504.7 ± 26.7 b
Transition	2489.1 ± 75.4 a	40.8 ± 3.2 a	393.0 ± 22.3 a	40.0 ± 2.8 a	1.2 ± 0.1 a	1.4 ± 0.3 a	14.2 ± 1.2 a	43.1 ± 2.7 a	17.0 ± 1.9 a	552.5 ± 25.5 a
Average	2357.5	37.3	378.8	35.0	1.1	1.3	13.8	38.6	15.7	493.0
RDA	2200	50	400	38	1.6	2	15	70	23	1000

Where; P—protein, C—carbohydrate, F—fat, T—thiamine, R—riboflavin, N—niacin, A—ascorbic acid, Fe—iron, Ca—calcium. Note: values in the column followed by different letter are statistically significant at *p* ≤ 0.05 level (N = 90, 30/zone ± SEm of quantities).

**Table 5:** Percent (%) contribution of wild edible foods (%) for energy and nutrients to diets consumed by indigenous communities of AABR, Central India.

Zone	Nutrients									
	Energy (Cal)	P (g)	C (g)	F (g)	T (mg)	R (mg)	N (mg)	A (mg)	Fe (mg)	Ca (mg)
Core	8.3 ± 0.8 a	4.9 ± 0.5 a	5.6 ± 0.4 a	13.5 ± 16 a	35.4 ± 3.2 a	8.4 ± 2.6 b	4.5 ± 0.5 a	73.0 ± 8.2 a	4.1 ± 1.8 a	26.7 ± 4.2 a
Buffer	6.5 ± 0.5 a	4.0 ± 0.7 a	5.0 ± 0.9 a	6.0 ± 1.3 b	28.9 ± 3.8 b	11.5 ± 2.5 a	4.2 ± 0.5 a	57.2 ± 9.2 b	5.4 ± 1.3 a	29.1 ± 3.8 a
Transition	3.7 ± 0.6 b	1.1 ± 0.6 b	3.2 ± 0.7 b	2.6 ±1.4 c	13.7 ± 4.2	2.4 ± 1.6 c	1.2 ± 0.4 b	38.1 ± 7.2 c	1.1 ± 2.1 b	17.2 ± 5.2 b
Mean	6.2	3.3	4.6	7.4	26.0	7.4	3.3	56.1	3.5	24.3

Where; P—protein, C—carbohydrate, F—fat, T—thiamine, R—riboflavin, N—niacin, A—ascorbic acid, Fe—iron, Ca—calcium; the values within columns with different letters statistically significant at *p* ≤ 0.05 level (N = 90; 30/zone ± SEm of percentage).

## Data Availability

Data could be made available on reasonable request from first author or corresponding author.

## Supplementary Materials

The following are available online at https://www.mdpi.com/article/10.3390/foods10071453/s1, Table S1: Diversified plant species found in Achanakmaar Amarkantak Biosphere Reserve (AABR) of Central India, and Table S2: Price list of important WEPs sold in hat (weekly) markets of AABR, Central India.
