# Supplementary material for: Use of Wild Edible Plants: Can They Meet the Dietary and Nutritional Needs of Indigenous Communities in Central India
Source: Foods. 2021 Jun 23;10(7):1453. doi: 10.3390/foods10071453 (PMC8307769; doi:10.3390/foods10071453)
Supplement: Supplementary file 1 [file foods-10-01453-s001.zip › foods-1204853-supplementary.pdf]

Table S1: Diversified plant species found in Achanakmaar Amarkantak Biosphere Reserve (AABR) of Central India

**Wild leafy edibles used by indigenous communities**										
S. No.	**Botanical name**	**Family**	**Local name**	**Habit**	**Habitat**	**Part used**	**Mode of utilization**	**Season of collection**	**Edible value**	**Diversity of uses**
1	*Achyranthes aspera* Linn.	Amaranthaceae	Chirchitta, Latjeera	H	Wasteland & Forest fringes	Tender Leaves	Cooked as vegetable	R	++	Medicinal and religious
2	*Alternanthera philoxeroides* Mart.	Amaranthaceae	Katuashak	H	Forest	Twigs and leaves	Cooked as vegetable	S	++	-
3	*Alternanthera sessilis* Mart.	Amaranthaceae	Garundi	H	Forests and wastelands lands	Twigs	Cooked as vegetable	S	++	Medicinal
4	*Amaranthus caudatus* Linn.	Amaranthaceae	Rajgirbhaji	H	Village commons	Seeds & Leaves	Cooked as vegetable	R	++++	-
5	*Amaranthus hybridus* Linn.	Amaranthaceae	Lalbhaji	H	Village commons	Leaves	Cooked as vegetable	W & S	++++	Medicinal
6	*Amaranthus spinosus* Linn.	Amaranthaceae	Kanta Cholai, Barre	H	Wastelands and fringes of forests	Leaves	Cooked as vegetable	R	+++	Medicinal
7	*Amaranthus viridis* Linn.	Amaranthaceae	Jungli chaulai	H	Wastelands and fringes of forests	Leaves	Cooked as Vegetable	R	++++	-
8	*Antidesma diandrum* Roxb.	Euphorbiaceae	Saroti	S	Forests	Leaves &fruits	Cooked as vegetable	S	+++	Medicinal & Firewood
9	*Argyreia strigosa* (Roth) Sant.	Convolvulaceae	Baghchooda	H	Wastelandslands	Young leaves	Cooked as vegetable	R	++	Medicinal
10	*Bambusa bambos* Linn.	Poaceae	Bans Kareel	S	Forests & Agricultural lands	Shoots	Vegetable, pickle	R	++++	Fodder, household articles etc.
11	*Bambusa vulgaris* Schrad.	Poaceae	Bans Kareel	S	Forests & Agricultural tural lands	Shoots	Vegetable, pickle	R	++++	Fodder, household articles etc.

12	*Basella alba* Linn.	Basellaceae	Ban Poi	Cl	Degraded forests	Leaves	Cooked as vegetable	W	+++	Medicinal
13	*Bauhinia purpurea* Linn.	Fabaceae	Koelar-Kachnar	T	Forests	Tender leaves & Flower buds	Cooked as vegetable	S	+++	Firewood & fodder. Bark dyes.
14	*Boerhavia diffusa* Linn.	Nyctigenaceae	Ghetuli	H	Forests	Leaves	Cooked as vegetable	R	++	Fodder & Medicinal
15	*Careya arborea* Roxb.	Myrtaceae	Kumbhibaj i	T	Degrade forests	Leaves	Cooked as vegetable	S	+++	Firewood, Medicinal
16	*Cassia fistula* Linn.	Fabaceae	Amltas, dhanbaher	T	Forest fringes	Flower buds and young leaves	Cooked with pulses	S	+++	Firewood, & Medicinal
17	*Cassia tora* Linn.	Fabaceae	Charota	H	Grassland and degrade forests	Young leaves	Leaves cooked as vegetable and roasted seeds eaten.	R	+++	Medicinal
18	*Celosia argentea* Linn.	Amaranthaceae	Phul Bhaji/Gadr ya	H	Wastelands and agriculture	Leaves	Cooked as vegetable	R	+++	-
19	*Chenopodium album* Linn.	Amaranthaceae	Batuabhaji	H	Agiculture wastelands	Leaves young shoots	Cooked as vegetable	W	++++	Medicinal
20	*Chlorophytum tuberosum* (Roxb.) Bak.	Asparagaceae	Safed Musli	H	Forests	Young leaves, bulbs	Cooked as vegetable	R & W	++	Medicinal
21	*Cleome viscosa* Linn.	Cleomaceae	Hurhurria	H	Wastelands	Leaves	Cooked as Vegetable	R & W	++	-
24	*Coccinia grandis* (Linn.) J. *Voigt*	Cucurbitaceae	Bendarikh and/Tendli	Cl	Wastelands	Young leaves &fruit	Cooked as Vegetable	S & W	++	-

22	*Colocasia esculenta* Linn.	Araceae	Jungle Arbi/ghuia	H	Swampy areas	Leaves & corms	Cooked as vegetable	S & W	+++	Medicinal
23	*Commelina benghalensis* Linn.	Commelinaceae	Kenna	H	Wastelands	Leaves & young shoots	Cooked as vegetable	R	++	Fodder & Medicinal
26	*Corchorus acutangulus* Lam.	Tiliaceae	Masaria Bhaji	H	Wastelands	Leaves	Cooked as vegetable	R	+++	-
25	*Corchorus trilocularis* Linn.	Tiliaceae	Chench	H	Wastelands	Leaves	Cooked as vegetable	R	+++	-
27	*Cordia myxa* Forst.	Boraginaceae	Bohar	T	Forest	Tender leaves & twigs	Cooked as vegetable and fruits pickled	S	+++	Firewood & Medicinal
28	*Dendrocalamus strictus* (Roxb.) Nees	Poaceae	Lathi bans	S	Forest & Agriculture lands	Young shoots and leaves	Cooked as vegetable & pickles	R	++++	Fodder, Live fencing, Fishing rods, agril implements household articles, Ladder, sticks, baskets
29	*Digera muricata* (L.) Mart	Amaranthaceae	Chanchali	H	Agriculture lands	Leaves	Cooked as vegetable	R & W	+++	Medicinal
30	*Emilia sonchifolia* (L.) DC.	Asteraceae	Hirankuri	H	Wastelands	Leaves & young shoots	Cooked as vegetable	R & W	++	Medicinal
31	*Eryngium foetidum* Linn.	Apiaceae	Van Dhania	H	Wastelands and forest	Leaves	Spice	R	+++	Medicinal
32	*Ficus infectoria* Roxb.	Moraceae	Pakri	T	Wastelands and forest	Leaves	Cooked as vegetable	Y	++	Fodder, firewood, Medicinal
33	*Hibiscus sabdariffa* L.	Malvaceae	Amari baji	H	Agriculture and homestaeds	Leaves	Cooked as vegetable	W	++++	-
34	*Hibiscus sp.*	Malvaceae	Bhalobhaji-Katta,	H	Agriculture lands	Leaves	Cooked as vegetable	W	+++	-

			Amarkatva, Patwa				& pickle			
35	*Ipomea aquatica* Forsk.	Convolvulaceae	Karmatta	Aq	Swampy areas	Leaves & twigs	Cooked as vegetable	S & W	++++	-
36	*Ipomea batatas* (Linn.) Lam.	Convolvulaceae	Kandabhaji Mitha Alu	H	Swampy areas	Young leaves & tubers	Cooked as vegetable, Kheer, Halua, Chips & boiled tubers	W	+++	-
37	*Ipomea eriocarpa* R. Br.	Convolvulaceae	Buta	H	Swampy areas	Young leaves	Cooked as vegetable	W	++	-
38	*Lagerstroemia parviflora* Roxb.	Lythraceae	Senna	T	Forest	Tender leaves	vegetable	S	+	Timber & Firewood
39	*Lepidium sativum* Linn.	Brassicaceae	Chandrasur	H	Forest and Agriculture lands	Young leaves	Cooked as vegetable	W	++	Medicinal
40	*Leucas aspera* (Willd) Linn.	Lamiaceae	Ghooma	H	Forest	Young leaves	Spice	R	++	-
41	*Limnophila rugosa* (Roth.) Merr.	Scrophulariaceae	Bintugo	H	Wastelands	Leaves	Cooked as vegetable	Y	+	Medicinal
42	*Marsilea minuta* Linn.	Marsileaceae	Sunsiniya	Aq	Agriculture and waste lands	Leaves	Cooked as vegetable	S & W	++++	Medicinal
43	*Mentha arvensis*	Lamiaceae	Pudina	H	Forest and Agril	Leaves	Spices & pickled	Y	++++	Medicinal
44	*Merremia emarginata* (Burm. F) Hallier.F	Convolvulaceae	Muskani Baji	H	Swampy areas	Leaves	Cooked as vegetable	R	+++	-
46	*Momordica charantia* Linn.	Cucurbitaceae	Karelabaji	Cl	Waste and Agriculture lands	Leaves & fruits	Cooked as vegetable	Y	+++	Medicinal
45	*Moringa oleifera* Lam.	Moringaceae	Munga, sahejan	T	Agriculture and village commons	Young leaves & pods	Cooked as vegetable	S & W	+++	Firewood & Medicinal
47	*Murraya koenigii* (Linn.) Sprengal	Rutaceae	Mitineem	T	Wastelands	Leaves	Spice additive	Y	+++	Medicinal
48	*Nelumbo nucifera*	Nelumbonaceae	Indian	Aq	Aquatic	Leaves	Cooked as	R	++	

	Gaertn.		lotus		bodies	and rhizomes	Vegetable			
49	*Olax scandens* Roxb.	Olaceae	Dhehiani	S	Forests	Leaves	Cooked as vegetable	R	+	-
50	*Oroxylum indicum* (Linn.) Benth.Ex Kurz	Bignoniaceae	Bhut-vriksha	T	Degraded forest	Leaves and stems	Cooked as vegetable	S & W	+	-
51	*Oxalis corniculata* Linn.	Oxalidaceae	Tinpania	Aq	Aquatic bodies	Leaves	Cooked as vegetable	S	+++	-
52	*Phyllocephalum indicum* (Less) Kirkman	Zingiberaceae	Vanjeera	H	Forests	Young leaves	Spice	R & W	+++	-
53	*Polygonum barbatum* Linn.	Polygonaceae	Bekhanjabaj	H	Forests and wastelands	Young leaves	Cooked as vegetable	R	+++	Medicinal
54	*Portulaca oleracea* Linn.	Portulaceae	Golbazi	H	Agriculture lands	Leaves	Cooked as vegetable	R & W	++++	-
55	*Portulaca quadrifida* Linn.	Portulaceae	Bhuibazi	H	Agriculture land	Leaves and young shoots	Cooked as vegetable	R	+++	Medicinal
56	*Rorippa indica* (Linn.) Hiern. Syn.	Brassicaceae	Chamsuru	H	Agriculture lands	Tender shoots & young leaves	Cooked as vegetable	W	++	-
57	*Shorea robusta* Gaertn.	Dipterocarpaceae	Hargami	T	Forests	Seeds and young leaves	Cooked as vegetable	S	+	Timber, NTFP &. Medicinal
58	*Smithia conferta* Sm.	Fabaceae	Duthi	H	Forests	Leaves	Cooked as vegetable	R	+	Medicinal
59	*Tamarindus indica* Linn.	Fabaceae	Imli	T	Agriculture and wastelands	Young leaves and flower buds	Cooked as vegetable	S	++	Firewood, Fruits-ripe fruits eaten, pulp Katta sag with pulses
60	*Trianthema potrulacstrum* Linn.	Aizoaceae	Salsa	H	Agriculture and	Young leaves	Cooked as vegetable	R	++	-

					wastelands					
**Wild flowers, fruits, seeds, nuts and pods used by indigenous communities**										
61	*Abelmoschus crinitus* Wall	Malvaceae	Kamlya	H	Flowers	Leaves and flowers	Forest Fringe s	W	+	Medicinal
62	*Abelomoshcus moschatus* Medik.	Malvaceae	Jungli bhendi	H	Fruits	Vegetable	Forest	W	+ +	Medicinal
63	*Adina cordifolia* (Roxb.) B & H	Rutaceae	Haldu, Karma	T	Fruit	Timber, Leaves and fruits	Forests	S	+	Timber, Medicinal uses
64	*Aegle marmelos* (L.) Corrêa	Rutaceae	Bel	T	Fruit	Cold beverarae	Wild and along forest fringe	S	+ + + +	Medicinal, Religious
65	*Ampelocissus barbata Wall.*	Vitaceae	Van angur	C	Berries	Ripe berries eaten	Forest	W	+ +	Folk medicine
66	*Annona squamosa* L.	Annonaceae	Sitaphal	S	Fruit	Ripe fruits eaten	Along forest fringes	W	+ + +	Medicinal
67	*Anogeissus latifolia* (Roxb. ex DC.) Wall.	Combretaceae	Dhaora/ dhawra	T	Leaves Bark	tanning and firewood	Forests	S	+	Medicinal, Gums
68	*Anthocephalous kadamba*	Rubiaceae	Kadam	T	Fruits	Ripe fruits eaten	Forest and open	W	+	-
69	*Artocarpus heterophylla Lam.*	Moraceae	Jackfruit	T	Fruit	Vegetable	Wild lands, Forests	S	+ + + +	Timber Medicinal, Religious
70	*Azadirachta indica* A.Juss.	Meliaceae	Neem	T	Fruit	Pulp of ripe fruits eaten	Wild Habita tion	R	+	M & Cultural
71	*Bahunia villi* (L.) Benth.	Fabaceae	Mahul	C	seeds	Roasted seeds are eaten	Forest, Agricu ltural & Wastel	W	+	Medicinal & NTFP

							ands			
72	*Bauhinia purpurea L.*	Fabaceae	Koelar	T	Floral buds	Floral buds cooked as vegetables	Forest	S	+ +	Medicinal
73	*Boeharavia diffusa L.*	Nyctaginaceae	Punarnava	H	Seeds	Eaten	Agricu ltural	W	+ +	Medicinal
74	*Bombax ceiba L.*	Malvaceae	Semal	T	Flowers	Cooked as vegetable	Forest	S	+	Silk cotton tree
75	*Bridelia retusa* (L.) A.Juss	Phyllanthaceae	Kaji, Kinu	T	Fruit, Bark, Flowers	Ripe fruit pulp	Forest	W	+	Medicinal
76	*Buchanania lanjan* Spreng.	Anacardiaceae	Char	T	Fruits & Seeds	Ripe fruits eaten & endosperm of seed	Forests	S	+ + + +	Edible and medicinal uses
77	*Butea monosperma* (Lam.) Taub.	Fabaceae	Parsa, Palas	T	Flower & Seed	Flower buds eaten	Wild lands & Forests	W	+	Medicinal
78	*Capparis zeylanicum L.*	Capparaceae		C	Fruits	Cooked as vegetable	Wild	S	+ +	Medicinal
79	*Carissa carandas L.*	Apocynaceae	Karonda	S	Fruits	Ripe fruits eaten & pickled	Agricu ltural	W	+ + + +	Live fence
80	*Cassia fistula L.*	Fabaceae	Amaltas	T	Flowers	Cooked	Forest	S	+ +	Medicinal
81	*Celastrus paniculatum* Willd.	Celastraceae	Malkangani	W C	FlowerYoun g	Flowers cooked as Vegetable	Forest	S	+	Medicinal
82	*Coccinia grandis Voigt.*	Cucurbitaceae	Van Kundru	C	Fruits	Unripe fruits as vegetable	Agricu ltural	Y	+ + +	Perennial climber, medicinal
83	*Coccinia indica*	Cucurbitaceae	Van Kundru	C	Fruits	Unripe fruits as vegetable	Agricu ltural	Y	+ +	Medicinal
84	*Cochlospermum religosum* (L.) Alston	Bixaceae	Kumbi	T	Flowers & Seeds	Flowers cooked as Vegetable	Forest	S	+	Medicinal
85	*Cordia dichotoma*	Boraginaceae	Lasooda	T	Fruits	Fruits cooked as	Forest	S	+	Medicinal

	G.Forst.					Vegetable and pickled			+ +	
86	*Dillenia pentagyna Roxb.*	Dilleniaceae	Karmal	T	Fruits	Immature fruits eaten and pickled	Forest	S	+	Medicine for Ayurveda and Folk
87	*Diospyros melanoxylon* Roxb.	Ebenaceae	Tendu	T	Fruit	Ripe fruits eaten	Forests	S	+ + +	Timber, NTFP, wrapping bidis
88	*Feronia elephantum* Corr.	Rutaceae	Kaith	T	Fruit	Ripe fruits eaten & pickled	Forest	S	+ + +	Edible food
89	*Ficus benghalensis L.*	Moraceae	Bargad	T	Fruit	Ripe fruits eaten	Wild lands, Forests	S	+ +	Religious
90	*Ficus hispida* L.f.	Moraceae	Kala Umbar	T	Fruits	Ripe fruits eaten. Unripe fruits vegetable	Wild lands	W	**+ +**	**Medicines**
91	*Ficus infectoria* (Miq.)	Moraceae	Pakar	T	Leaves	Cold beverarae	Forest and wildla nds	W	+ +	Medicinal
92	*Ficus religiosa L.*	Moraceae	Pipal	T	Fruit	Ripe fruits eaten	Wild lands, Forests	S	+ +	Religious
93	*Flacourtia indica (Burm.f.) Merr.*	Salicaceae	Alu Bukhara	T	Fruit	Fruit - raw or cooked	Forest Agricu ltural, and open	W	+	Sweet, Jam, Jelly, Wine, Medicinal
94	*Flacourtia indica* (Burm.f.) Merr.	Salicaceae	Bilangada	S, T	Fruits	Raw eaten or cooked	Forest fringes	S	+	Herbal medicine
95	*Grewia asiatica L.*	Malvaceae	Phalsa	T	Fruit	The fruit can be eaten raw,	Forest	W	+ +	Edible, Medicinal
96	*Grewia rothii DC.*	Malvaceae	Kiraman	S	Bark	Medicinal	Open Forest area	S	+ +	Cultural, Medicinal, NTFPs

97	*Indigofera pulchela L.*	Fabaceae	Neeli	H	Flower	Vegetable	Agriculture	S	+ +	Medicinal
98	*Lantana camara L.*	Verbenaceae	Raimuniya	S	Fruits	Ripe fruits are eaten	Forest & Agriculture	S	+ +	Medicinal
99	*Limonia crenulata (Roxb.) Roem*	Rutaceae	Beli	S	Fruit	Eaten	Forest	W	+	Medicinal
100	*Madhuca indica* (Roxb.) A.Chev.	Sapotaceae	Mahua	T	Flower Unripe fruits, seed	Flowers and fruits raw eaten, beverage, seeds oil	Forests & Agriculture lands	S	+ + + +	Medicinal & Religious
101	*Mangifera indica* L.	Anacardiaceae	Am	T	Ripe and unripe Fruit	Ripe fruits eaten & unripe pickled	Forest & Agricultural lands	S	+ + + +	Rituals & Cultural
102	*Meyna spinosa Roxb.*	Rubiaceae	Munya	S	Fruit	Raw eaten	Forest	W	+	Medicine
103	*Mimuopsis elengi L.*	Sapotaceae	Maulshree	T	Fruits	Ripe fruits are eaten	Habitation	S	+	Medicinal
104	*Momordica charantia*	Cucurbitaceae	Van karela	C	Unripe	Cooked as vegetables	Agriculture & Forest fringes	R	+ + + +	Medicinal
105	*Momordica dioica, Spreng.*	Cucurbitaceae	Jungli keksi	C	Unripe Fruits	Cooked as vegetables	Agriculture & Forest fringes	R	+ + +	Medicinal
106	*Moringa oleifera*	Moringaceae	Munga	T	Leaves, flowers & pods	Cooked as vegetables	Agriculture & Forest fringes	R	+ + + +	Medicinal
107	*Morus alba L.*	Moraceae	Sahtoot	T	Fruits	Ripe fruits are eaten	Agriculture	W	+ + +	Traditional medicine
108	*Mucuna pruriens*	Fabaceae/ Leguminosae	Kewanch	C	Pods	Pods cooked as vegetable	Wild lands	W	+ +	M

							and forest fringes			
109	*Passiflora foetidia L*	Passifloraceae	Jhumka lata	C	Fruits	Ripe fruits are eaten	Agricultural lands	W	+	M
110	*Phoenix sylvestris* (L.) Roxb.	Arecaceae	Wild kajur	T	Fruit	Ripe fruits eaten	Agriculture	R	+ +	Medicinal (Fruit/Seed /Root), Food, Fodder, Fuelwood
111	*Phyllanthus emblica L.*	Phyllanthaceae	Aonla	T	Fruits	Ripe fruits eaten & pickled	Forest & Agricultural lands	W	+ + + +	Medicinal & Rituals
112	*Pithecolobium dulce* (Roxb.) Benth.	Caesal pinaceae	Gangaimli	T	Fruit	Aril of fruits eaten	Wild lands, Forests	W	+ + +	Traditional medicine
113	*Prosopis cineraria*	Fabaceae	Jungli sami, Chikur, Khejri	T	Fruit	Leaves and fruit eaten	Forest and Open	S	+	Medicinal & NTFPs
114	*Schleichera oleosa, Oken*	Sapindaceae	Kusum	T	Fruits	Ripe fruits eaten	Forest	S	+ +	NTFP, Lac production
115	*Semecarpus anacardium Linn.*	Anacardiaceae	Bheluva	T	Fruits& seeds	Roasted fruits are eaten	Degraded forests	S	+ + +	Medicinal. Marking of clothes
116	*Sesbania grandiflora* (L.) Pers.	Fabaceae	Agathi	T	Flower & tender fruits	Vegetable	Agriculture	Y	+ +	Medicinal
117	*Shorea robusta Roth.*	Dipterocarpaceae	Sarai/Sal	T	Seeds, Leaves	Oil from seeds	Forest fringes	W	+ +	Biodiesel
118	*Solanum nigrum, Linn*	Solanaceae	Makai	H	Fruits	Ripe fruits eaten	Wild lands	W	+ +	Medicinal
119	*Sterculia urens Roxb.*	Sterculiaceae	Karaya	T	Seeds and gum	Seeds – roasted	Forest	S	+	NTFP Gum edible thicker and

										binding of foods
120	*Strychnos nuxvomica L.*	Loganiaceae	Kuchla	T	Fruits	Eaten by children	Forest	S	+	Water purifier
121	*Syzigium cumini* (L.) Skeels	Myrtaceae	Jamun	T	Fruit	Ripe fruits eaten, syrup	Forests & wild lands	S	+ + + +	Medicinal
122	*Tamarindus indica L.*	Leguminosae	Imli	T	Flowers, fruits and seeds	Flower as vegetable, fruits eaten and seeds flour	Forest & Agricu ltural lands	S	+ + + +	Medicinal
123	*Terminalia bellerica Roxb.*	Combretaceae	Bahera	T	Fruit	Ripe fruits eaten	Forests	S	+ +	Medicinal & Timber
124	*Tetrastigma lanceolarium Roxb.*	Vitaceeae	Bharseri	C	Berries	Ripe berries eaten	Forest		+	Edible and medicinal
125	*Trichosanthes cucumerina L.*	Cucurbitaceae	Jungli chichoda	C	Fruit	Eaten	Forest and Open	S	+	Medicinal
126	*Woodfordia floribunda* (L.). Kurz.	Lytharaceae	Dhavai	S	Flowers	Flowers are eaten as food	Forest	S	+	Medicinal
127	*Ziziphus jujuba Mill.*	Rhamnaceae	Ber	T	Fruits	Ripe fruits are eaten	Wild lands, Forests	W	+ + +	Medicinal and Religious
128	*Ziiyphus martuiana Lam.*	Rhamnaceae	Chota ber	S	Fruits	Ripe fruits are eaten	Wild lands & Forests	W	+ + +	Medicinal and Religious
129	*Ziziphus oenophylla Lam.*	Rhamnaceae	Makora	S	Fruits	Eaten by children	Forest	W	+ +	Medicinal
130	*Ziziphus xylopyrus* (Retz.) Willd.	Rhamnaceae	Kathber, Baraber	S	Fruits	Eaten and used as drug	Forest	W	+	Medicinal
**Wild edible rhizomes, roots, underground stems and tubers used by indigenous communities**										
131	*Amorphophallus bulbifera*	Araceae	Zamin	H	Corn	Corn is edible	Forest	W&S	++	Medicina

	(Roxb.) Bl.		kand			after boiling.				l
132	*Amorphophallus paeoniifolius* (Dennst.) N.	Araceae	Suran kand	H	Corn	Corms, and stem eaten and cooked as vegetable.	Habitations and Agriculture lands	W&S	+++	Medicinal
133	*Asparagus racemosus* Willd.	Liliaceae	Shatavari,	H	Tuber, root	Tubers are consumed as vegetable	Forest	W& S	++	Medicinal
134	*Chlorophytum arundinaceum* (Roxb.) Baker	Asparagaceae	Safed musli	H	Suckers	Root	Forest and agricultural land	R	++	Medicinal
135	*Chlorophytum tuberosum (Roxb.) Baker*	Asparagaceae	Musli	H	Suckers	Root	Forest and agricultural land	R	++	Medicinal
136	*Colocasia esculentum L.*	Araceae	Kochai	H	Tuber	Corms, petiole and leaves are cooked as vegetable.	Agriculture lands	W	++++	-
137	*Costus speciosus* (J.Konig) C.Specht	Zingiberaceae	Kewkanda	H	Rhizomes	Rhizomes eaten as vegetable, chutney and pickle.	Forest	W	++	Medicinal
138	*Curculigo orchioides Gaertn*.	Hypoxidaceae	Kalimusli	H	Tuber	Tuberous roots eaten as vegetables	Forest	W&S	++	Medicinal
139	*Curcuma angustifolia L.*	Zingiberaceae	Thikur	H	Rhizome	Strach extracted from rhizome Rhizomes as Sarbat, Halwa and Barfi	Forest	W	+++	Medicinal
140	*Curcuma caesia* Roxb	Zingiberaceae	Kalihaldi	H	Rhizomes	Rhizomes used as Spice and flavour ing.	Agriculture lands	W	++	Medcinal
141	*Cyperus esculent Linn*	Cyperaceae	Nagar motha	H	Tuber	Eaten tubers	Swamps	R, W	++	Medicinal
142	*Dioscorea alata (L.)*	Dioscoreaceae	Nagarkand, Uskakand	C	tuber	Raw tubers used as vegetable	Forest	W	+++	Medicines

143	*Dioscorea bulbifera* (L.)	Dioscoreaceae	Damgkand a	C	tuber	Aerial and tubers used as vegetable and boiled form for consumption.	Forest	W	+++	Medicina l
144	*Dioscorea hispida Dennst.*	Dioscoreaceae	Kuliakand	C	tuber	Tubers eaten as chips	Forest	W	+++	-
145	*Dioscorea pentaphylla L.*	Dioscoreaceae	Suwarkan da,	C	tuber	Tubers cooked as vegetable.	Forest	W	+++	Medicina l
146	*Dioscorea pubera Blume*	Dioscoreaceae	Ban Alu	C	Tuber	Eaten	Forest	W	++	-
147	*Ipomoea cairica (L.) Sweet*	Convovulaceae	Neeli Bel	C	Tuber	Leaves cooked and eaten as a vegetable Tubers & stem cooked.	Swamps	W	++	-
148	*Leea macrophylla* (Roxb. ex Hornem) H	Leeaceae	Dhotelaka nd	H/S	Stems, Leaves, roots	Roots cooked as vegetable	Forest	R	+++	Medicina l
149	*Nelumbium nucifera Gaerth*	Nelumbonaceae	Kamal	H	Rhizomes, stem, tuber	Root cooked as a vegetable	Wetlands	R, W	+++ +	-
150	*Pueraria tuberosea* (Roxb. ex Willd) C	Fabaceae	Patal kumda Bidari kand	H	Tuber	Tubers root cooked as vegetable.	Forest	R	+++	Medicina l
151	*Scirpus grossus* (L.f.) e G	Cyperaceae	Kaseru Kand	H	Tubers	Tubers consumed as vegetable	Wetlands	W	+	-
152	*Trapa nutans L.*	Lythraceae	Singhada	H	Tubers	Seeds are raw and boiled eaten	Wet lands	W	+++ +	-
153	*Urginea indica* (Roxb.) Kunth	Liliaceae	Jangli-piyaz	H	Bulb	Bulbs cooked as vegetable	Forest	W	++	Medicina l
**Wild edible mushrooms used by indigenous communities**										
S. No.	**Botanical name**	**Family**	**Local name**	**Part used**	**Mode of utilization**		**Habitat**	**Season of collectio**	**Edi ble val**	**Diversit y of uses**

							**n**	**ue**	
154	*Agaricus compestris* L.	Agaricaceae	Butter mushroom	Fruiting body	Cooking as Vegetable, Used as flore	Agriculture, degraded & wild lands	June-August	++++	Edible
155	*Astraeus asiaticus Phosri, Martín & Watling*	Diplocystaceae	Puttu	Fruiting body	Vegetable, pickle, papad & medicine	Forests, Wasteland, degraded lands	June-August	++++	Edible, Medicinal
156	*Astraeus hygrometricus (Pers.) Morgan*	Diplocystaceae	Puttu, Rugra	Fruiting body	Vegetable, pickle, papad & medicine	Wasteland, wild lands, degraded	June-August	+++	Edible, Medicinal
157	*Astraeus koreanus (Pers.) Morgan*	Diplocystaceae	Puttu	Fruiting body	Vegetable, pickle, papad & medicine	Forests, Wasteland, wild lands, degraded land	June-August	+++	Edible, Medicinal
158	*Astraeus morganii (Pers.) Morgan*	Diplocystaceae	Puttu	Fruiting body	Vegetable, pickle, papad & medicine	Wasteland, Forests, degraded land	June-August	+++	Edible, Medicinal
159	*Astraeus odoratus Phosri, Watling, Martín & Whalley*	Diplocystaceae	Puttu	Fruiting body	Vegetable, pickle, papad & medicine	Wasteland, wild lands, degraded	June-August	+++	Edible, Medicinal
160	*Astraeus smithii Watling, Martín & Phosri*	Diplocystaceae	Puttu	Fruiting body	Vegetable, pickle, papad & medicine	Wasteland, wild lands, degraded	June-August	+++	Edible, Medicinal
161	*Ganoderma lucidum (Curtis) Karst*	Polyporaceae Or Ganodermataceae	Reishi mushroom	Fruiting body	Medicine	Dense Sal Forest, Wild land, Forests	July-October	+++	Medicinal
162	*Lepiota procera (Scop. ex Fr.) Kumm.*	Paxilliacaeae	Parasol	Caps	Vegetable	Forest fringes, Agriculture	July-August	+++	Edible, Medicinal
163	*Macrolepiota dolichaula Singer*	Agaricaceae	Parasol	Caps and stem	Cooking as vegetable	Forest fringes, Agriculture	July-August	+++	Edible, Medicinal
164	*Pleurotus flabellatus Berk. & Br*	Tricholomataceae	Oyester	Fruiting body	Vegetable, pickle, papad, flore & medicine	Forest fringes, Agriculture, open areas,	July-August	++++	Edible, Medicinal
165	*Russula congoana Pat.*	Russulaceae	Sarai Pihri	Fruiting body	Medicine & Cooking	Dense Sal forest, Forest fringes	June-August	++	Medicinal

166	*Russula solaris Ferd. & Winge*	Russulaceae	Sarai Pihri	Fruiting body	Cooking	Forest fringes, Open land	June - August	++	Edible, Medicinal
167	*Scleroderma bovista Fr.*	Agaricaceae	Potato Pihri	Fruiting body	Vegetable	Forests, degraded & wild lands, grassland	July-August	+++	Edible, Medicinal
168	*Termitimyces clypeatus R. Heim*	Lyophyllaceae	Pihri-Chirko	Fruiting body	Vegetable & pickle	Forest fringes, forest villages	July - August	++++	Edible, Medicinal
169	*Termitimyces heimii Natarajan*	Lyophyllaceae	Sarai Pihri	Fruiting body	Medicine & cooking	Dense Sal forest, Forest fringes	July - August	++	Medicinal
170	*Termitomyces eurhizus (Berk.) R. Heim*	Lyophyllaceae	Pihri - Bhondo	Fruiting body	Vegetable & pickle	Forest fringes, forest villages	July - August	++++	Edible, Medicinal
171	*Termitomyces microcarpus (Berk. & Broome)*	Lyophyllaceae	Pihri -Bhat	Fruiting body	Vegetable & pickle	Forest fringes, forest villages	July - August	++++	Edible, Medicinal
172	*Termitomyces sp R. Heim*	Lyophyllaceae	Pihri- Raj Bhondo	Fruiting body	Vegetable, pickle, papad & medicine	Forest fringes, forest villages	July - August	++++	Edible, Medicinal

Where; T- Tree, S- Shrub, H-Herb, Aq- Aquatic, Cl-climber, R-Rainy, W-Winter, S- Summer, Y-throughout the year, ++++-Very high, +++-High, ++ - Moderate, +-Least, NTFP-Non timber forest product

Table S2: Price list of important WEPs sold in *hat* (weekly) markets of AABR, Central India

S. No.	Botanical name	Family	Local name	Unit	Price (Rs.)
1	*Aegle marmelos (L.) Corrêa* (F & D)	Rutaceae	Bel	*1 kg*	*15-20*
2	*Annona squamosa L.* (F)	Annonaceae	Sitaphal	*12 nos*	*40-60*
3	*Madhuca indica (Roxb.) A.Chev.* (D)	Sapotaceae	Mahua	*1 kg*	*30-40*
4	*Diospyros melanoxylon Roxb.* (F)	Ebenaceae	Tendu	*1 kg*	*20-25*
5	*Syzigium cumini (L.) Skeels* (F)	Myrtaceae	Jamun	*1 kg*	*30-40*
6	*Feronia elephantum Corr.* (F)	Rutaceae	Kaith	*1 kg*	*25-30*
7	*Mangifera indica L.* (F)	Anacardiaceae	Am	*1 kg*	*30-80*
8	*Phyllanthus emblica* L. (F & D)	Phyllanthaceae	Aonla	*1 kg*	*40-60*
9	*Tamarindus indica* L. (D)	Leguminosae	Imli	*1 kg*	*25-40*
10	*Buchanania lanjan Spreng.* (F & D)	Anacardiaceae	Char	*1 kg*	*150-200*
11	*Zizyphus jujuba Mill.* (F & D)	Rhamnaceae	Ber	*1 kg*	*30-40*
12	*Pithecolobium dulce (Roxb.) Benth.* (F)	Caesal pinaceae	Gangaimli	*1 kg*	*25-30*
13	*Artocarpus heterophylla Lam.* (F)	Moraceae	Jackfruit	*1 kg*	*10-15*
14	*Carissa carandas L.* (F)	Apocynaceae	Karonda	1 kg	20-25
15	*Terminalia bellerica Roxb.* (D)	Combretaceae	Bahera	1 kg	25-30
16	*Bauhinia purpurea L.* (F)	Fabaceae	Koelar	1 kg	20-25
17	*Coccinia grandis Voigt.* (F)	Cucurbitaceae	Van Kundru	1 kg	10-20

18	*Coccinia indica* (F)	Cucurbitaceae	Van Kundru	1 kg	50-70
19	*Momordica dioica, Spreng.* (F)	Cucurbitaceae	Jungli keksi	1 kg	15-25
20	*Momordica charantia* (F)	Cucurbitaceae	Van karela	1 kg	15-20
21	*Moringa oleifera* (F)	Moringaceae	Munga	1 kg	20-30
22	*Shorea robusta Roth.* (D)	Dipterocarpaceae	Sarai/Sal	1 kg	20-30
23	*Cordia dichotoma* G.Forst. (F)	Boraginaceae	Lasooda	1 kg	80-100
24	*Woodfordia floribunda* (L.). Kurz. (D)	Lytharaceae	Dhavai	1 kg	25-30
25	*Sterculia urens Roxb.* (D)	Sterculiaceae	Karaya	1 kg	40-70
26	*Strychnos nuxvomica L.* (D)	Loganiaceae	Kuchla	1 kg	20-30
27	*Achyranthes aspera* Linn. (F)	Amaranthaceae	Chirchitta,	1 kg	10-20
28	*Amaranthus caudatus* Linn. (F)	Amaranthaceae	Rajgirbhaji	1 kg	10-20
29	*Amaranthus hybridus* Linn. (F)	Amaranthaceae	Lalbhaji	1 kg	10-20
30	*Amaranthus spinosus* Linn. (F)	Amaranthaceae	Kanta Cholai,	1 kg	10-20
31	*Amaranthus viridis* Linn. (F)	Amaranthaceae	PurpuriBhaji/	1 kg	10-20
32	*Bambusa bambos* Linn. (F)	Poaceae	Bans Kareel	1 kg	20-30
33	*Bambusa vulgaris* Schrad. (F)	Poaceae	Bans Kareel	1 kg	20-30
34	*Basella alba* Linn. (F)	Basellaceae	Ban Poi	1 kg	20-25
35	*Bauhinia purpurea* Linn. (F)	Fabaceae	Koelar-Kachnar	1 kg	10-15
36	*Cassia tora* Linn. (F)	Fabaceae	Charota	1 kg	10-15

37	*Chlorophytum tuberosum* (Roxb.) Bak. (F)	Asparagaceae	Safed Musli	1 kg	20-35
38	*Colocasia esculenta* Linn. (F)	Araceae	Jungle Arbi/ghuia	1 kg	60-70
39	*Cordia myxa* Forst. (F)	Boraginaceae	Bohar	1 kg	20-30
40	*Dendrocalamus strictus* (Roxb.) Nees (F)	Poaceae	Lathi bans	1 kg	25-35
41	*Ipomea aquatica* Forsk. (F)	Convolvulaceae	Karmatta	1 kg	10-20
42	*Marsilea minuta* Linn. (F)	Marsileaceae	Sunsiniya	1 kg	10-15
43	*Merremia emarginata* (Burm. F) Hallier. (F)	Convolvulaceae	Muskani Baji	1 kg	10-15
44	*Nelumbo nucifera Gaertn.* (F)	Nelumbonaceae	Indian lotus	1 kg	25-30
45	*Portulaca oleracea* Linn. (F)	Portulaceae	Golbazi	1 kg	15-20
46	*Mentha arvensis* (F)	Lamiaceae	Pudina	1 kg	15-20
47	*Hibiscus sabdariffa* L. (F)	Malvaceae	Amari baji	1 kg	10-15
48	*Amorphophallus paeoniifolius* (Dennst.) (F)	Araceae	Suran kand	1 kg	20-30
49	*Amorphophallus bulbifera (Roxb.) Bl.* (F)	Araceae	Zamin kand	1 kg	15-20
50	*Asparagus racemosus* Willd. (F)	Liliaceae	Shatavari,	1 kg	20-30
51	*Curcuma angustifolia L.* (F&D)	Zingiberaceae	Thikur	1 kg	30-45
52	*Colocasia esculentum L.* (F)	Araceae	Kochai	1 kg	20-30
53	*Dioscorea bulbifera* (L.) (F)	Dioscoreaceae	Damgkanda	1 kg	15-25
54	*Dioscorea pentaphylla L.* (F)	Dioscoreaceae	Suwarkanda,	1 kg	15-25
55	*Dioscorea alata (L.)* (F)	Dioscoreaceae	Nagarkand,	1 kg	15-25

56	*Dioscorea hispida Dennst.* (F)	Dioscoreaceae	Kuliakand	1 kg	15-25
57	*Dioscorea pubera Blume* (F)	Dioscoreaceae	Ban Alu	1 kg	15-25
58	*Nelumbium nucifera Gaerth*	Nelumbonaceae	Kamal	1 kg	25-35
59	*Chlorophytum arundinaceum (Roxb.)* (F&D)	Asparagaceae	Safed musli	1 kg	35-40
60	*Chlorophytum tuberosum (Roxb.) Baker* (F)	Asparagaceae	Musli	1 kg	40-50
61	*Trapa nutans L.* (F)	Lythraceae	Singhada	1 kg	120-150
62	*Termitomyces sp R. Heim (*D&F)	Lyophyllaceae	Raj Bhondo	1 kg	100-120
63	*Termitomyces microcarpus (Berk. Broome)* (F&D)	Lyophyllaceae	Pihri –Bhat	1 kg	100-110
64	*Termitomyces eurhizus (Berk.) R. Heim (*D&F)	Lyophyllaceae	Pihri – Bhondo	1 kg	80-100
65	*Termitimyces clypeatus R. Heim(*D&F)	Lyophyllaceae	Pihri- Chirko	1 kg	70-90
66	*Astraeus asiaticus Phosri,* (D&F)	Diplocystaceae	Puttu	1 kg	100-130
67	*Agaricus compestris L. (*D&F)	Agaricaceae	Butter mushroom	1 kg	90-120
68	*Pleurotus flabellatus Berk. & Br*(D&F)	Tricholomataceae	Oyester	1 kg	100-125
69	*Anogissus latifolia* (Roxb. ex DC.) Wall. (D)	Lythraceae	Dhaura	1 kg	40-80
70	*Murraya koenigii* (Linn.) Sprengal (F & D)	Rutaceae	Mitineem	1 kg	30-50
71	*Abelmoschus moschatus* Medik. (D)	Malvaceae	Katuri Bhendi	1 kg	30-40
72	*Oxalis corniculata* Linn. (F)	Oxalidaceae	Tinpatiya	1 kg	15-25

Note: INR Rs. 73 = 1 US Doller; D for Dry component and F for Fresh.
